# Supplementary material for: Phosphorylation-mimicking histone H3.3 rescues exercise-induced gene responses in an epigenetic aging model of mouse skeletal muscle
Source: Lab Anim Res. 2025 Sep 24;41:25. doi: 10.1186/s42826-025-00254-6 (PMC12459025; doi:10.1186/s42826-025-00254-6)

Re-submitted to Laboratory Animal Research on June 12, 2025.

**Phosphorylation-mimicking histone H3.3 rescues exercise-induced gene responses in an epigenetic aging model of mouse skeletal muscle**

Sho Maruyama, and Fuminori Kawano

Graduate School of Health Science, Matsumoto University, Nagano, Japan

Corresponding author:

Fuminori Kawano, Ph.D.

Graduate School of Health Science, Matsumoto University

2095-1 Niimura, Matsumoto City, Nagano 390-1295, Japan.

Phone: +81-263-48-7372, Fax: +81-263-48-7290

e-mail: kawano@t.matsu.ac.jp

**Abstract**

Background: With aging, the canonical histone H3.1/3.2 in skeletal muscle is progressively replaced by the non-canonical variant H3.3. Although H3.3 is thought to be involved in age-related epigenetic regulation due to its role as a histone variant, its functional characteristics remain largely unknown. Serine 31 (S31) is a unique amino acid residue of H3.3 that undergoes phosphorylation. Therefore, the present study aimed to investigate the relationship between skeletal muscle aging and H3.3 phosphorylation at S31 (H3.3S31ph).

Results: We first demonstrated that H3.3S31ph levels were significantly reduced in the tibialis anterior muscle of 75-wk-old mice compared to 8-wk-old mice. We then examined the effects of viral vector–mediated expression of wild-type H3.3 or a phosphorylation-mimicking H3.3 mutant (H3.3S31E) on gene responsiveness to acute exercise in aging skeletal muscle. In muscles expressing wild-type H3.3, which simulates epigenetic alterations observed during skeletal muscle aging, the transcriptional response to acute exercise was lost by 30 weeks post-treatment (60 weeks of age). In contrast, expression of H3.3S31E successfully rescued the gene responses to acute exercise. This rescue was accompanied by increased enrichment of H3K4me3 and H3K27me3 following acute exercise in the H3.3S31E group, whereas no such histone modification changes were observed in the wild-type H3.3 group. Additionally, robust involvement of exogenous H3.3 in exercise-related histone turnover was observed in the wild-type H3.3 group, but not in the H3.3S31E group, suggesting that phosphorylation at S31 limits the dynamic behavior of H3.3.

Conclusions: Impaired transcriptional responsiveness to exercise in a simulated epigenetic aging model induced by exogenous H3.3 expression was rescued by the phosphorylation-mimicking H3.3S31E variant in middle-aged skeletal muscle. The findings of the present study demonstrate that H3.3S31ph plays a critical role in regulating the stability of H3.3 within chromatin.

Keywords: epigenetics, aging, histone modifications, histone variant, mutant histone

**Background**

Sarcopenia is a progressive condition characterized by the age-related loss of skeletal muscle mass and function. This process is driven by multiple factors, including motor neuron loss, mitochondrial dysfunction, immune dysregulation, and oxidative stress-induced neuromuscular impairment [[1](#_ENREF_1), [2](#_ENREF_2)]. While the prevalence of sarcopenia increases markedly in individuals aged 70 years and older, previous studies [[3](#_ENREF_3), [4](#_ENREF_4)] have demonstrated that a decline in skeletal muscle mass begins as early as the fourth decade of life in men. This suggests that age-related skeletal muscle changes might be initiated during earlier stages of adulthood. Consequently, a prolonged "pre-symptomatic state" of skeletal muscle aging likely precedes the eventual decline in overall muscular function observed in advanced age. However, the specific alterations that occur in skeletal muscle during this pre-symptomatic period remain poorly understood.

DNA methylation and histone modifications, as components of the epigenetic regulatory system, are closely associated with aging. Belsky et al. [[5](#_ENREF_5), [6](#_ENREF_6)] reported that in cohort studies investigating variations in the pace of aging, DNA methylation patterns in blood cells correlated with physical and cognitive function, as well as subjective signs of aging. These correlations were observed with biological age, which was estimated based on 18 biomarkers reflecting organ-system integrity among individuals born in the same year. In the context of skeletal muscle aging, age-associated DNA methylation patterns have been identified through comparisons between young and elderly individuals [[7](#_ENREF_7)]. Masuzawa et al. [[8](#_ENREF_8)] reported that age-related replacement of canonical histone H3.1/3.2 with the non-canonical variant H3.3 positively correlates with H3K27me3 levels in the tibialis anterior muscle of mice. Furthermore, they found that the tibialis anterior muscle of middle-aged mice failed to upregulate exercise-related gene expression in response to acute running exercise, potentially due to the formation of repressive chromatin mediated by the co-occupancy of H3.3 and H3K27me3. Although these epigenetic alterations contribute to the age-related decline in skeletal muscle function, the precise role of H3.3 replacement in skeletal muscle aging remains largely unknown.

While canonical histones H3.1 and H3.2 are predominantly expressed during early developmental stages, non-canonical histone H3.3 gradually replaces them with age [[9](#_ENREF_9)]. The rate of age-related replacement of H3.1/3.2 with H3.3 varies among tissue types: in mice, H3.3 levels relative to H3.1/3.2 plateau by 10 months of age in the kidney and brain, whereas H3.3 levels in the heart continue to increase until 24 months of age [[10](#_ENREF_10)]. Harada et al. [[11](#_ENREF_11)] reported that histone H3.3 is essential for myogenic differentiation, as its incorporation into the loci of myogenic markers, such as myogenin and myosin heavy chain, was associated with increased expression of these genes and active H3 turnover. Furthermore, Esteves de Lima et al. [[12](#_ENREF_12)] demonstrated that satellite cell-specific knockout of histone chaperone HIRA led to insufficient incorporation of H3.3 into the regulatory regions of muscle genes, impairing the maintenance of H3K27ac modification. This resulted in reduced satellite cell self-renewal and diminished muscle regenerative capacity. These findings underscore the essential role of H3.3 incorporation into nucleosomes in myogenesis and muscle regeneration. Serine 31 (S31) is a unique amino acid residue of H3.3 that undergoes phosphorylation. Protein phosphorylation adds negative charge to amino acid side chains, and negatively charged amino acids, such as aspartic acid and glutamic acid, can sometimes mimic the phosphorylated state of a protein [[13](#_ENREF_13)]. The previous study utilizing phosphorylation-mimicking models demonstrated that H3.3S31 phosphorylation (H3.3S31ph) inhibits KDM4B, a demethylase targeting H3K9me3 and H3K36me3, thereby regulating heterochromatin accessibility at telomeres in mouse embryonic stem cells [[14](#_ENREF_14)]. Martire et al. [[15](#_ENREF_15)] demonstrated that depletion of H3.3 from mouse embryonic stem cells reduced acetylation on histone H3K27 at enhancer regions, along with reduced ability to reprogram cell fate. However, exogenous expression of H3.3S31E that mimics phosphorylation at S31 recovered the reduced H3K27 acetylation and increased the expression of differentiation-specific genes. These findings suggest that H3.3S31ph serves as a key modulator of H3.3-specific function. However, age-related alterations in H3.3S31ph and its role in skeletal muscle aging remain unknown. Therefore, the present study aimed to investigate the relationship between aging and H3.3S31ph in mouse skeletal muscle. To achieve this, we utilized a viral vector to induce the expression of a mutant H3.3 that mimics phosphorylation at H3.3S31.

**Methods**

*Ethical approval and animal care*

All experimental procedures were performed in accordance with the Guide for the Care and Use of Laboratory Animals of Matsumoto University (Nagano, Japan). Animal experiments (approval ID: 2023-3 and 2024-3) and genetic modification experiments (approval ID: 2023-2 and 2024-2) were approved by the Animal Use Committee and Genetic Modification Safety Committee in Matsumoto University, respectively. Additionally, all experimental procedures were confirmed to comply with the ARRIVE Guidelines 2.0 (https://arriveguidelines.org/arrive-guidelines).

Male C57BL/6J mice at 7, 29, 74 weeks of age were purchased from CLEA Japan (Tokyo, Japan) and used for the present study. Mice were acclimated to the experimental environment for 1 week before being used in the experiments described below. A commercial solid diet (CE-2, CLEA Japan) and water were supplied ad libitum. Temperature and humidity in the animal room were maintained at 23°C and 40-60%, respectively, with a 12:12 h light-dark cycle.

*Experimental design*

The mice at 8-wk-old and 75-wk-old (n=3 each) were used for the comparison of histone protein levels between young and aged mice. The mice at 30-wk-old (n=54) were used for the aging study with a viral vector administration. The mice were separated into the Stuffer, H3.3, and H3.3S31E groups (n=18 each) and the administration of various viral vectors was performed at 30-wk-old. At 40-, 50-, and 60-wk-old, the mice were further separated into the control and exercise groups (n=3 each) in each treatment with viral vector. The mice in the exercise group were assigned to perform a single bout of exercise. The present study aimed to investigate the effects of enhanced phosphorylation at H3.3S31 during aging, a period when H3.3 replacement is also elevated in skeletal muscle. Although expression of the phosphorylation-mimicking H3.3S31E variant can reproduce the functional consequences of S31 phosphorylation, interpretation of its effects requires comparison with a wild-type H3.3 expression model. This is necessary to account for the baseline effects induced by H3.3 expression itself, which also serves to simulate epigenetic aging in skeletal muscle. In addition, the wild-type H3.3 model allows for evaluation of the relationship between the amount of H3.3 and its phosphorylation status.

*Design and administration of viral vector*

Adeno-associated virus serotype 9 (AAV9) vector was modified to encode H3.3A (gene symbol: *H3f3a*) or its phosphorylation-mimicking mutant H3.3S31E at the downstream of ACTA1 promoter (VectorBuilder Japan, Kanagawa, Japan). For constructing H3.3S31E mutant, the codon TCT encoding S31 was replaced with GAA (E31). As for the control group, the *H3f3a* sequence was replaced with untranslatable 249 bp sequence (stuffer). AAV9 vector was intramuscularly injected into both right and left tibialis anterior muscles under inhalation anesthesia by Isoflurane. Single shot injection (50μL) was performed to administer 1×10^11^ viral genomes per muscle.

*Acute exercise*

The mice in the exercise group (n=3 in each treatment with viral vector) performed a total of 30 minutes of running on an animal treadmill (Panlab Harvard Apparatus, Barcelona, Spain), starting at a speed of 15 cm/s, gradually increasing to 20 cm/s, and then to 25 cm/s. The tibialis anterior muscles were sampled from both control and exercise groups 2 hours post-exercise. Muscle sampling was performed immediately after carbon dioxide euthanasia. Each mouse was placed in an inhalation chamber and exposed to increasing concentrations of carbon dioxide. Excess fat and connective tissue were removed from the muscle tissue, which was then frozen in liquid nitrogen and stored at −80°C until further analysis.

*Western blotting*

Total histones were extracted using the Epiquik Total Histone Extraction Kit (Epigentek, Farmingdale, NY, USA). Muscle samples weighing 20 mg were processed by adding 500 µL of lysis buffer provided in the kit. The samples were then centrifuged at 12,000g for 5 minutes at 4 °C, and 300 µL of the resulting supernatant was carefully collected. This supernatant was then mixed with 90 µL of balance buffer supplied in the kit. The histone extract was further combined with an equal volume of 2 × SDS sample buffer (containing 20% glycerol, 12% 2-mercaptoethanol, 4% sodium dodecyl sulfate, 100 mM Tris–HCl, and 0.05% bromophenol blue, pH 6.7) and boiled for 10 minutes.

Western blotting was performed as described previously [[16](#_ENREF_16)]. The following antibodies were used to detect each protein: HA tag (ab9110, Abcam, Cambridge, UK, 1: 1000), H3.3 (ab176840, Abcam), H3.1/3.2 (61629, Active Motif, Carlsbad, CA, USA, 1: 1000), H3K4me3 (9751, Cell Signaling Technology, Danvers, MA, USA, 1: 1000), H3K9me3 (61013, Active Motif, 1: 1000), H3K27me3 (9733, Cell Signaling Technology, 1: 1000), H3.3S31ph (ab92628, Abcam, 1: 1000), total H3 (4620, Cell Signaling Technology, 1: 1000), total CHK1 (37010, Cell Signaling Technology), CHK1 phosphorylated at serine 296 (pCHK1) (90178, Cell Signaling Technology), H3T3ph (9714, Cell Signaling Technology), H3S10ph (53348, Cell Signaling Technology), and H3S28ph (9713, Cell Signaling Technology). The antibody-bound proteins were detected using a chemiluminescence technique with Western BLoT Hyper HRP Substrate (Takara Bio Inc., Shiga, Japan) using ChemiDoc Touch MP (Bio-Rad, Hercules, CA, USA). The bands were quantified using image analysis software (ImageJ) (https://imagej.net/ij/). The protein level was expressed as the integrated density of the band, which was calculated as the mean density multiplied by the band area. Finally, the integrated density was compared between the experimental groups that were applied to the same membrane.

*RNA extraction*

A frozen muscle sample (20 mg) was homogenized in 1 mL of ISOGEN (NIPPON GENE, Toyama, Japan) following the manufacturer's instructions for total RNA extraction. Total RNA (800 ng) was then used to synthesize cDNA using SuperScript VILO Master Mix (Thermo Fisher Scientific, Waltham, MA, USA). The mixture samples were incubated at 42°C for 60 minutes, followed by enzyme inactivation at 85°C for 5 minutes. The synthesized cDNA was then diluted to a 1/10 concentration with ultrapure water and stored at −20°C until analysis.

*Chromatin immunoprecipitation (ChIP)*

Extraction of chromatin-rich extract and ChIP reaction were performed in accordance with the previously described methods [[17](#_ENREF_17)]. Briefly, frozen muscle sample (20 mg) were homogenized in cooled PBS. After centrifugation at 12,000g, the pellet was fixed in 1% paraformaldehyde on ice for 10 minutes followed by quenching in 200 mM glycine. The pellet was resuspended in lysis buffer and sonicated using a Sonifier 250 (Branson, Danbury, CT, USA). Because it was necessary to obtain an average DNA fragment size of 500 bp for ChIP-qPCR analysis, sonication was repeated four times. After centrifugation at 12,000g, the supernatant was further gel-filtrated to remove small DNA fragments and free histones, which did not form nucleosomes, and stored as the chromatin-rich extract at −80 °C until analysis. Chromatin-rich extracts containing equal DNA content (500 ng) were combined within each group and used for the ChIP reaction. Chromatin was reacted with antibody: HA tag (9110, Abcam, 1: 50), H3K4me3 (9751, Cell Signaling Technology, 1: 50), H3K9me3 (13969, Cell Signaling Technology, 1: 50), H3K27me3 (9733, Cell Signaling Technology, 1: 50), and total H3 (4620, Cell Signaling Technology, 1: 50) for 1 h at 4 °C, followed by a reaction with Dynabeads™ Protein A (Thermo Fisher Scientific, Waltham, MA, USA) for 30 minutes at 4 °C. Beads were washed and incubated with proteinase K (Takara Bio Inc.) for 1 h at 65 °C. DNA was extracted and resuspended in Tris-EDTA buffer and stored at −20 °C. ChIP rection using same antibody was tested twice to minimize the differences between reactions, and the yielded DNA was combined within each group. The level of input DNA contained in chromatin used for each ChIP reaction was also tested without any reactions.

*Quantitative PCR (qPCR)*

qPCR was performed using the StepOne Real-Time PCR System (Thermo Fisher Scientific). THUNDERBIRD NEXT SYBR qPCR Mix (Toyobo, Osaka, Japan) was used for PCR, following the manufacturer’s recommended dilution procedures. For gene expression analysis, a set of 20 genes, previously identified to show increased expression in the tibialis anterior muscle following acute running exercise, was utilized [[18](#_ENREF_18)]. For ChIP-qPCR, the primer pairs were designed at 1 kbp upstream and downstream of the transcription start site (TSS) for each of the 20 genes [[18](#_ENREF_18)]. The downstream region of *Ppargc1a* locus was excluded from the ChIP-qPCR analysis, since an insufficient primer amplification, likely due to instability in nucleosome formation, was observed.

For gene expression assay, quantification of the qPCR results was performed by normalizing the cycle threshold (Ct) values of target amplification. *Gapdh* mRNA was used as an internal control. Ct values from each biological replicate were measured by qPCR, and the normalized data were averaged within each experimental group (n=3 each group). To standardize the data range across genes, the averaged value was further converted into a relative value, with the mean set to 1 for each gene. This relative value was then used as a data point for each gene in the dot plots. For ChIP-qPCR analysis, the chromatin samples from three biological replicates were pooled for each ChIP reaction. The qPCR results were normalized by the Ct values of respective input samples (% input). The normalized % input values were further converted into relative values for each gene and used as data points in the dot plots.

*Statistical analysis*

Statistical analysis was performed using BellCurve for Excel (Social Survey Research Information, Tokyo, Japan). Significant difference was examined by one-way ANOVA followed by Scheffe’s post hoc test. Student’s unpaired t test was used to compare the two groups. Pearson correlation was used to test the significance of the strength and direction of association between two factors. Differences were considered significant at p < 0.05.

**Results**

*Age-related changes in H3.3S31ph*

We first examined the differences in the histone modifications between young (8-wk-old) and aged mice (75-wk-old). The level of H3.3 relative to H3.1/3.2 in the tibialis anterior muscle was significantly increased (3-fold) with age (Fig. 1A), consistent with the findings of our previous study [[8](#_ENREF_8)]. Despite the increase in H3.3, the level of H3.3S31ph was decreased by −34% at 75-wk-old compared to 8-wk-old (p<0.05) (Fig. 1A). Although we previously reported a positive correlation between H3.1/3.2 and H3K4me3 levels, as well as between H3.3 and H3K27me3 levels [[8](#_ENREF_8)], no significant differences in H3K4me3 or H3K27me3 levels were observed in the present study (Fig. 1A). This may suggest that the absolute differences in these histone modifications are too small to be detected through a simple comparison between only two timepoints.

CHK1 [[19](#_ENREF_19)] and AURK-B [[20](#_ENREF_20)] are known upstream regulatory kinases responsible for H3.3S31 phosphorylation. Previous study [[15](#_ENREF_15)] reported that inhibition of CHK1, but not AURK-B, effectively reduced H3.3S31ph levels in mouse embryonic stem cells. Based on this finding, we re-analyzed histone-rich extracts of tibialis anterior muscle obtained in our previous study [[8](#_ENREF_8)] to assess age-dependent changes in CHK1 phosphorylation and H3.3S31ph. The level of H3.3S31ph significantly declined with age (−30% at 32-wk-old vs 8-wk-old, −42% at 53-wk-old vs 32-wk-old, and −77% at 75-wk-old vs 53-wk-old), with the most pronounced decrease occurring between 53 and 75 weeks of age (Fig. 1B).　Both pCHK1 and total CHK1 levels were maintained up to 53-wk-old but declined at 75-wk-old (−68%, p=0.066, and −63%, p=0.009, vs 8-wk-old, respectively) (Fig. 1B). In contrast, phosphorylation at other histone residues (H3T3, H3S10, and H3S28) remained unchanged with age (Fig. 1B). Significant correlations were observed between H3.3S31ph levels and both pCHK1 and total CHK1 levels (Fig. 1C).

*Effects of viral vector administration on histone modifications*

Based on the observation that aged mice exhibited a decrease in H3.3S31ph levels, we next examined the effects of forced H3.3 expression, with or without a phosphorylation-mimicking mutation at S31, during the aging period. HA tag was consistently detected in both H3.3 and H3.3S31E groups by western blotting throughout the experimental period, indicating the successful induction of vector-derived H3.3 (Fig. 2A-C). However, the level of H3.3 remained unchanged in both the H3.3 and H3.3S31E groups compared to the Stuffer group (Fig. 2A-C). Although the H3K9me3 level increased in the H3.3S31E group at 40- and 50-wk-old (+55%, p=0.117 and +61%, p<0.05 vs. Stuffer, respectively), this change was not observed at 60-wk-old (Fig. 2A-C). No significant changes were observed in H3.3S31ph, H3K4me3, and H3K27me3 levels throughout the experimental period (Fig. 2A-C). In terms of H3.3S31ph levels, the H3.3S31E group showed lower expression at 40- and 50-wk-old (−28%, p = 0.688 and −29%, p = 0.286 vs. H3.3 group, respectively), whereas an increase was observed at 60-wk-old (+32%, p = 0.179 vs. H3.3 group).

*Responses of genes to acute exercise*

To clarify the effects of forced H3.3S31E expression on muscle function, we used acute exercise as an evaluation model to examine the gene expression and epigenetic responses. The responsiveness of genes to exercise was assessed by analyzing a set of 20 target genes, as previously described [[18](#_ENREF_18)]. These genes were originally identified as being transcriptionally upregulated 2 hours after acute treadmill running in mice, based on RNA sequencing analysis, and their significance was subsequently confirmed by qPCR. We also previously reported that this gene set was responsive to exercise in the tibialis anterior muscle of young mice, but not in middle-aged mice [[8](#_ENREF_8)]. At 40-wk-old, acute exercise significantly upregulated the expression of target genes in all groups (+70%, +78%, +98% vs. control in Stuffer, H3.3, and H3.3S31E groups, respectively) (Fig. 3). A similar upregulation was observed in the Stuffer (+122% vs. control) and H3.3S31E (+119% vs. control) groups at 50-wk-old (p<0.001), although the H3.3 group showed significantly less upregulation compared to the other groups (+69% vs. control, p<0.05). Exercise-induced upregulation of target gene expression was maintained until 60-wk-old in the Stuffer (+83% vs. control) and H3.3S31E (+59% vs. control) groups, whereas no significant response to acute exercise was observed in the H3.3 group.

Since the effects of forced H3.3S31E expression on the gene responses to exercise were most pronounced at 60-wk-old, ChIP-qPCR analysis was conducted using muscle samples from 60-wk-old mice. The distribution of the HA tag at both upstream and downstream regions from TSS of the loci that were targeted to analyze the gene expression was significantly increased at resting levels in the control groups of exogenous H3.3 and H3.3S31E-expressing muscles, indicating that the viral vector-induced histones were successfully incorporated into nucleosomes (Fig. 4A). The distribution of HA tag further increased in response to acute exercise at both upstream and downstream regions of TSS in the H3.3 group, but not in the H3.3S31E group. Acute exercise significantly increased the distribution of H3.3 at both upstream and downstream regions of TSS in the Stuffer (+52% and +37% vs. control, respectively) and H3.3 (+51% and +60% vs. control, respectively) groups (Fig. 4B). However, in the H3.3S31E group, the magnitude of the increase in the H3.3 distribution post-exercise was +29% at the downstream region of TSS compared to the control level (p=0.008), while no significant differences were observed at the upstream region. The total H3 distribution was significantly increased after acute exercise at both upstream and downstream regions of TSS in the Stuffer (+51% and +42% vs. control, respectively) and H3.3S31E (+72% and +79% vs. control, respectively) groups (Fig. 4C). In contrast, no changes in total H3 distribution were observed after acute exercise in the H3.3 group.

Acute exercise significantly upregulated H3K4me3 at both upstream and downstream regions of TSS in the Stuffer (+75% and +138% vs. control, respectively) and H3.3S31E (+148% and +107% vs. control, respectively) groups (Fig. 4D). However, H3K4me3 at the target loci did not respond to acute exercise in the H3.3 group. The distribution of H3K9me3 was significantly upregulated at the downstream region of TSS in the control group of H3.3S31E-expressing muscle compared to the Stuffer and H3.3 groups (+64% and +47%, respectively) (Fig, 4E). At these sites, H3K9me3 was significantly downregulated (−33% vs. control) by acute exercise in the H3.3S31E group, whereas the distribution of H3K9me3 was upregulated (+63% vs. control, p<0.05) in the Stuffer group. H3K9me3 showed no response to acute exercise in the H3.3 group. A drastic upregulation in H3K27me3 distribution was observed at both upstream (+88% vs. control, p<0.001) and downstream (+84% vs. control, p<0.001) regions of TSS in response to acute exercise in the H3.3S31E group (Fig. 4F), whereas acute exercise had no effect on the distribution of H3K27me3 in the Stuffer or H3.3 groups.

To estimate the rate of histone turnover, ChIP-qPCR results for HA tag, H3.3, and total H3 were used to calculate the relative values of HA tag/H3.3 and H3.3/total H3. The HA tag/H3.3 ratio reflects the relative amount of exogenously expressed H3.3. This ratio was comparable between the H3.3 and H3.3S31E groups under basal conditions (Fig. 5A). After exercise, the HA tag/H3.3 ratio at the upstream regions of TSS significantly increased in the H3.3 group (+49% vs. control), while no change was observed in the H3.3S31E group. In contrast, at the downstream regions of TSS, the HA tag/H3.3 ratio remained unchanged after exercise in the H3.3 group, but significantly decreased in the H3.3S31E group (−29% vs. control). The H3.3/total H3 ratio represents the proportion of H3.3 within total H3 and was significantly decreased at both the upstream (−29% vs. Stuffer) and downstream (−27% vs. Stuffer) regions of TSS in the H3.3 group under basal conditions (Fig. 5B). This decrease was not observed in the H3.3S31E group. Following exercise, the H3.3/total H3 ratio remained unchanged in the H3.3 group, whereas it was significantly reduced in the H3.3S31E group at both upstream (−28% vs. respective control) and downstream (−27% vs. respective control) regions of the TSS.

**Discussion**

*Age-related-changes in H3.3S31ph*

The results of the present study demonstrated that H3.3S31 phosphorylation was downregulated with age in mouse tibialis anterior muscle. We previously reported age-related replacement of canonical histone H3.1/3.2 with the non-canonical variant H3.3 in mouse tibialis anterior muscle, which was associated with changes in histone modifications [[8](#_ENREF_8)]. For accurate quantification of H3.3 levels by western blotting, normalization to the corresponding H3.1/3.2 level is necessary. Total H3 may not be suitable as a normalization control, as the antibody may preferentially detect H3.3 or H3.1/3.2. Although the absolute level of H3.3 appears unchanged between 8- and 75-week-old mice in the present data (Fig. 1), consistent with our previous study [[8](#_ENREF_8)], age-associated replacement of H3.1/3.2 with H3.3 was also observed. Therefore, the current results suggest that the increase in H3.3 relative to H3.1/3.2 does not directly lead to phosphorylation at serine 31.

H3.3S31ph gradually declined with age, with the most pronounced decrease observed between 53 and 75 weeks of age (Fig. 1B). During this same life stage, both phosphorylated and total CHK1 levels also decreased, suggesting that not only CHK1 activity (as reflected by phosphorylation) but also the total CHK1 protein level contribute to maintaining H3.3S31ph during aging. Furthermore, both phosphorylated and total CHK1 levels were significantly correlated with H3.3S31ph levels across the aging timeline (Fig. 1C), indicating that CHK1 plays a continuous role in regulating H3.3S31 phosphorylation throughout aging. CHK1 is known to be essential for cell proliferation and survival by playing a critical role in the progression of the G2/M phase of the cell cycle [[21](#_ENREF_21)]. However, because myonuclei are in a post-mitotic state, it is unlikely that CHK1 is upregulated within myonuclei, even in the skeletal muscle of young individuals. This implies that the proliferative activity of satellite cells, key myogenic precursors that contribute new myonuclei during growth [[22](#_ENREF_22)], may serve as a major regulatory source of CHK1 phosphorylation during early life. Masuzawa et al. [[8](#_ENREF_8)] demonstrated that the number of myonuclei in the tibialis anterior muscle is significantly reduced in middle-aged mice compared to young mice, suggesting that the satellite cell-mediated supply of new myonuclei declines with age. This observation further supports the idea that satellite cell proliferation and their fusion into muscle fibers may contribute to CHK1 expression in skeletal muscle during growth, thereby maintaining CHK1 phosphorylation in early life.

*Experimental model for epigenetic aging*

The current experimental model for forced H3.3S31E expression was based on an exogenous H3.3 expression system, thereby functioning as a mutant histone expression model built upon H3.3 replacement that mimics age-related epigenetic alterations in skeletal muscle. However, in the present study, the total H3.3 level in the H3.3 group remained unchanged, as demonstrated by both western blotting (Fig. 2) and ChIP analysis (Fig. 4B). This suggests that age-related replacement with endogenous H3.3 may have masked the increase in H3.3 resulting from exogenous expression. Another possible explanation for the undetectable increase in H3.3 levels in both the H3.3 and H3.3S31E groups is that free histones that are not assembled into nucleosomes are more susceptible to degradation. Indeed, although H3.3 mRNA levels decreased during the normal aging process in skeletal muscle, its protein levels increase relative to the canonical variants H3.1/3.2 with age [[8](#_ENREF_8)], indicating that H3.3 replacement occurs in a quantity-independent manner. Nevertheless, the incorporation of HA-tagged H3.3 into nucleosomes in both the H3.3 and H3.3S31E groups (Fig. 4A) confirmed successful delivery and chromatin integration of the exogenous H3.3, indicating that it effectively replaced endogenous H3.3 in nucleosome assembly. Furthermore, the results demonstrated that exogenous H3.3 expression alone did not induce detectable changes in histone modification levels. Since the H3.3S31ph-specific antibody does not recognize the H3.3S31E mutant, the slight reduction in H3.3S31ph levels observed at 40- and 50-wk-old (Fig. 2A and B) may reflect competitive inhibition of endogenous H3.3S31 phosphorylation by exogenous H3.3S31E. In contrast, the slight increase in H3.3S31ph at 60-wk-old in the H3.3S31E group (Fig. 2C) may indicate a relative decline in H3.3S31ph levels in the H3.3 group. Our previous study [[8](#_ENREF_8)] reported that during normal aging, the relative increase in H3.3 correlated positively with H3K27me3, whereas forced expression of H3.3 via viral vector in young skeletal muscle led to a reduction in H3K27me3. These inverse findings suggest that changes in histone modifications such as H3K27me3 are not solely dependent on H3.3 abundance, but are more strongly influenced by age-related upstream regulatory mechanisms. Taken together, the current model of exogenous H3.3 expression may represent an early replacement of canonical histones H3.1/3.2 with H3.3 in middle-aged muscle, thereby simulating epigenetic aspects of skeletal muscle aging.

*Effects of forced H3.3S31E expression under basal conditions*

Although HA tag levels were increased at the target loci in the H3.3 group (Figs. 4A and 5A), the H3.3/total H3 ratio was decreased under basal conditions compared to the Stuffer group (Fig. 5B), indicating that H3.3 turnover was enhanced by exogenous H3.3 expression. In contrast, the H3.3/total H3 ratio was maintained in the H3.3S31E group under the same conditions (Fig. 5B), suggesting that expression of the phosphorylation-mimicking H3.3S31E variant decelerated the H3.3 turnover otherwise promoted by exogenous H3.3. These results suggest that H3.3S31 phosphorylation may play a role in suppressing histone dynamics, potentially by stabilizing H3.3 within nucleosomes.

H3K9me3 and H3K27me3 are hallmark histone modifications of heterochromatin, which is characterized by a condensed and transcriptionally repressive chromatin state. Constitutive heterochromatin, marked by H3K9me3, typically forms at gene-poor regions such as pericentromeres and telomeres, whereas H3K27me3 is enriched in facultative heterochromatin, which silences gene transcription in a context-dependent manner [[23](#_ENREF_23), [24](#_ENREF_24)]. In the present study, expression of H3.3S31E led to increased H3K9me3 levels at the downstream regions of TSS under basal conditions (Fig. 4E). However, while this increase was also observed at the whole-tissue level by western blotting at 50 weeks of age, it was no longer evident at 60 weeks (Fig. 2). As reported previously [[14](#_ENREF_14)], H3.3S31 phosphorylation promotes H3K9me3 enrichment. Conversely, we previously demonstrated that H3K9me3 levels decline with age in skeletal muscle, in parallel with reductions in H3.1/3.2 levels [[8](#_ENREF_8)]. These opposing influences may act in a compensatory manner, ultimately masking the H3.3S31E-induced increase in H3K9me3 when assessed at the total protein level by western blotting at 60 weeks. The localized accumulation of H3K9me3 observed at the downstream regions of the TSS in the H3.3S31E group may instead reflect reduced histone turnover, as discussed in the previous section. Taken together, these findings suggest that while H3.3S31 phosphorylation promotes H3K9me3 enrichment, the persistence of this mark may depend on the local rate of histone turnover. Furthermore, H3K4me3, a histone modification associated with transcriptional activation [23], was significantly decreased (−60%) at the upstream regions of the TSS in the H3.3S31E group compared to the H3.3 group (Fig. 4D). Although the mechanistic link between H3K4me3 and histone turnover remains unclear, these findings imply that H3.3S31 phosphorylation may differentially influence chromatin structure at upstream versus downstream regions of TSS, even under basal, resting conditions in skeletal muscle.

*Role of H3.3S31ph in gene responses to exercise*

The enhanced H3.3 turnover at the target loci observed in the H3.3 group resulted in a failure of gene responsiveness to exercise at 60-wk-old (Fig. 3). In the Stuffer group, acute exercise led to an upregulation of H3K4me3, a histone modification associated with transcriptional activation [23], at both upstream and downstream regions of TSS (Fig. 4D), which likely contributed to the exercise-induced increase in gene expression. In contrast, the H3.3 group failed to induce changes in H3K4me3 levels in response to exercise. Interestingly, the H3.3 group showed a significant increase in HA tag enrichment at the loci following exercise (Fig. 4A), while the total H3 distribution remained unchanged (Fig. 4C). This indicates that exogenous H3.3 was preferentially involved in histone turnover in response to exercise. On the other hand, the H3.3S31E group exhibited no change in HA tag distribution after exercise, despite showing a similar increase in total H3 levels. These results suggest that in the H3.3S31E group, non-H3.3 histone variants, such as H3.1 or H3.2, were preferentially utilized during transcriptional activation. The relatively decreased H3.3 distribution at the target loci observed after exercise in the H3.3S31E group (Fig. 5B) further supports the notion that histone turnover involving H3.3 was suppressed in this group. These findings collectively indicate that phosphorylation at serine 31 restricts H3.3 dynamics, thereby limiting its availability for exercise-induced histone turnover.

We previously reported that both acute and chronic exercise increased H3K27me3 levels at exercise-responsive gene loci in the tibialis anterior muscle of mice [[16](#_ENREF_16), [25](#_ENREF_25)]. Shimizu et al. [[25](#_ENREF_25)] further demonstrated that intramuscular overexpression of the H3K27 methyltransferase EZH1 via viral vector markedly enhanced both H3K4me3 and H3K27me3 levels in response to acute exercise, resulting in augmented gene responsiveness and training adaptation. In the present study, similar increases in H3K4me3 and H3K27me3 levels were observed in the H3.3S31E group following acute exercise (Fig. 4D and F), suggesting that this bivalent histone modification plays a key role in preserving transcriptional responsiveness to exercise under epigenetically aged conditions. Interestingly, in the H3.3S31E group, acute exercise appeared to shift the chromatin state at the downstream region of TSS from a single accumulation of H3K9me3 at rest, to a bivalent modification involving both H3K4me3 and H3K27me3 after exercise. At the upstream region of TSS, H3K9me3 levels were relatively high compared to H3K4me3, largely due to a reduction in baseline H3K4me3 levels. These findings suggest that the prevalence of H3K9me3 may regulate H3.3 dynamics during exercise-induced gene activation, as discussed in the previous section. Furthermore, the current results indicate that the deposition of H3K4me3 and H3K27me3 failed to occur when histone turnover predominantly involved H3.3 in response to exercise. In contrast, these modifications were deposited when H3.3S31E was present at the loci. This raises the possibility that exercise-induced deposition of H3K4me3 and H3K27me3 may preferentially occur on canonical histones, such as H3.1 and H3.2, or alternatively, on H3.3 only when phosphorylated at serine 31.

**Conclusions**

The present study investigated the relationship between skeletal muscle aging and H3.3S31ph. In the tibialis anterior muscle of mice, H3.3S31ph levels were significantly downregulated with age, whereas the ratio of H3.3 to canonical histones H3.1/3.2 increased significantly. We further examined the effects of forced expression of a phosphorylation-mimicking H3.3 mutant (H3.3S31E) via viral vector during the aging process. In muscles expressing wild-type H3.3, which simulates the epigenetic alterations observed in aged skeletal muscle, the transcriptional response to acute exercise was lost by 30 weeks post-treatment (at 60 weeks of age). In contrast, expression of H3.3S31E successfully rescued the exercise-induced gene responses. This rescue was accompanied by enhanced enrichment of H3K4me3 and H3K27me3 following acute exercise in the H3.3S31E group, whereas no such histone modification changes were observed in the H3.3 group. Additionally, exogenous H3.3 was robustly involved in histone turnover in response to acute exercise in the H3.3 group, but not in the H3.3S31E group, suggesting that phosphorylation at S31 limits the dynamic behavior of H3.3. Taken together, our findings demonstrate that H3.3S31 phosphorylation plays a critical role in regulating the stability of H3.3 within chromatin. The age-related decline in H3.3S31ph likely enhances H3.3 turnover, thereby interfering with the deposition of histone modifications in response to exercise in skeletal muscle.

**List of abbreviations**

AURK-B, aurora kinase B

CHK1, checkpoint kinase 1

EZH1, enhancer of zeste homolog 1

H3K27ac, histone H3 acetylated at lysine 27

H3K27me3, histone H3 trimethylation at lysine 27

H3K36me3, histone H3 trimethylation at lysine 36

H3K4me3, histone H3 trimethylation at lysine 4

H3K9me3, histone H3 trimethylation at lysine 9

H3S10ph, histone H3 phosphorylated at serine 10

H3S28ph, histone H3 phosphorylated at serine 28

H3T3ph, histone H3 phosphorylated at threonine 3

HIRA, histone regulator A

KDM4B, lysine demethylase 4B

**Declarations**

Ethics approval and consent to participate: Animal experiments (approval ID: 2023-3 and 2024-3) and genetic modification experiments (approval ID: 2023-2 and 2024-2) were approved by the Animal Use Committee and Genetic Modification Safety Committee in Matsumoto University, respectively.

Consent for publication: Not applicable.

Availability of data and materials: The datasets used and/or analysed during the current study are available from the corresponding author on reasonable request.

Competing interests: The authors declare that they have no competing interests.

Funding: The present study was supported by JSPS KAKENHI Grant Numbers 21H03383 (FK), 23K21634 (FK) and 21K18296 (FK), and The Uehara Memorial Foundation (FK).

Authors' contributions: FK conceptualized and designed the study. SM and FK performed animal experiments. SM analyzed the muscle samples. SM and FK wrote the draft of manuscript. SM and FK read and approved the final manuscript.

Acknowledgements: Not applicable.

**References**

1. Yuan S, Larsson SC. Epidemiology of sarcopenia: Prevalence, risk factors, and consequences. Metabolism. 2023;144:155533.

2. Wiedmer P, Jung T, Castro JP, Pomatto LCD, Sun PY, Davies KJA, et al. Sarcopenia - Molecular mechanisms and open questions. Ageing Res Rev. 2021;65:101200.

3. Kelly TL, Wilson KE, Heymsfield SB. Dual energy X-Ray absorptiometry body composition reference values from NHANES. PLoS One. 2009;4(9):e7038.

4. Lee MM, Jebb SA, Oke J, Piernas C. Reference values for skeletal muscle mass and fat mass measured by bioelectrical impedance in 390 565 UK adults. J Cachexia Sarcopenia Muscle. 2020;11(2):487-96.

5. Belsky DW, Caspi A, Arseneault L, Baccarelli A, Corcoran DL, Gao X, et al. Quantification of the pace of biological aging in humans through a blood test, the DunedinPoAm DNA methylation algorithm. Elife. 2020;9.

6. Belsky DW, Caspi A, Corcoran DL, Sugden K, Poulton R, Arseneault L, et al. DunedinPACE, a DNA methylation biomarker of the pace of aging. Elife. 2022;11.

7. Voisin S, Seale K, Jacques M, Landen S, Harvey NR, Haupt LM, et al. Exercise is associated with younger methylome and transcriptome profiles in human skeletal muscle. Aging Cell. 2024;23(1):e13859.

8. Masuzawa R, Rosa Flete HK, Shimizu J, Kawano F. Age-related histone H3.3 accumulation associates with a repressive chromatin in mouse tibialis anterior muscle. J Physiol Sci. 2024;74(1):41.

9. Maehara K, Harada A, Sato Y, Matsumoto M, Nakayama KI, Kimura H, et al. Tissue-specific expression of histone H3 variants diversified after species separation. Epigenetics Chromatin. 2015;8:35.

10. Tvardovskiy A, Schwammle V, Kempf SJ, Rogowska-Wrzesinska A, Jensen ON. Accumulation of histone variant H3.3 with age is associated with profound changes in the histone methylation landscape. Nucleic Acids Res. 2017;45(16):9272-89.

11. Harada A, Maehara K, Sato Y, Konno D, Tachibana T, Kimura H, et al. Incorporation of histone H3.1 suppresses the lineage potential of skeletal muscle. Nucleic Acids Res. 2015;43(2):775-86.

12. Esteves de Lima J, Bou Akar R, Machado L, Li Y, Drayton-Libotte B, Dilworth FJ, et al. HIRA stabilizes skeletal muscle lineage identity. Nat Commun. 2021;12(1):3450.

13. Pearlman SM, Serber Z, Ferrell JE, Jr. A mechanism for the evolution of phosphorylation sites. Cell. 2011;147(4):934-46.

14. Udugama M, Vinod B, Chan FL, Hii L, Garvie A, Collas P, et al. Histone H3.3 phosphorylation promotes heterochromatin formation by inhibiting H3K9/K36 histone demethylase. Nucleic Acids Res. 2022;50(8):4500-14.

15. Martire S, Gogate AA, Whitmill A, Tafessu A, Nguyen J, Teng YC, et al. Phosphorylation of histone H3.3 at serine 31 promotes p300 activity and enhancer acetylation. Nat Genet. 2019;51(6):941-6.

16. Shimizu J, Kawano F. Exercise-induced histone H3 trimethylation at lysine 27 facilitates the adaptation of skeletal muscle to exercise in mice. J Physiol. 2022;600(14):3331-53.

17. Kawano F, Nimura K, Ishino S, Nakai N, Nakata K, Ohira Y. Differences in histone modifications between slow- and fast-twitch muscle of adult rats and following overload, denervation, or valproic acid administration. J Appl Physiol (1985). 2015;119(10):1042-52.

18. Ohsawa I, Kawano F. Chronic exercise training activates histone turnover in mouse skeletal muscle fibers. FASEB J. 2021;35(4):e21453.

19. Chang FT, Chan FL, JD RM, Udugama M, Mayne L, Collas P, et al. CHK1-driven histone H3.3 serine 31 phosphorylation is important for chromatin maintenance and cell survival in human ALT cancer cells. Nucleic Acids Res. 2015;43(5):2603-14.

20. Chan FL, Vinod B, Novy K, Schittenhelm RB, Huang C, Udugama M, et al. Aurora Kinase B, a novel regulator of TERF1 binding and telomeric integrity. Nucleic Acids Res. 2017;45(21):12340-53.

21. Tang J, Erikson RL, Liu X. Checkpoint kinase 1 (Chk1) is required for mitotic progression through negative regulation of polo-like kinase 1 (Plk1). Proc Natl Acad Sci U S A. 2006;103(32):11964-9.

22. Murach KA, White SH, Wen Y, Ho A, Dupont-Versteegden EE, McCarthy JJ, et al. Differential requirement for satellite cells during overload-induced muscle hypertrophy in growing versus mature mice. Skelet Muscle. 2017;7(1):14.

23. Saksouk N, Simboeck E, Dejardin J. Constitutive heterochromatin formation and transcription in mammals. Epigenetics Chromatin. 2015;8:3.

24. Trojer P, Reinberg D. Facultative heterochromatin: is there a distinctive molecular signature? Mol Cell. 2007;28(1):1-13.

25. Shimizu J, Horii N, Ono Y, Kawano F. EZH1 as a key mediator of exercise-induced H3K27me3 and H3K4me3 in mouse skeletal muscle. Adv Exer Health Sci. 2024;1(4):270-8.

**Figure legends**

Figure 1: Age-related changes in histone levels. (A) H3.1/3.2, H3.3, H3.3S31ph, H3K4me3, H3K27me3, and total H3 were analyzed by western blotting using histone-rich extracts from the tibialis anterior muscle. Representative blot images from 8-wk-old and 75-wk-old mice are shown. H3.3 levels are presented as the ratio relative to H3.1/3.2. The levels of H3.3S31ph, H3K4me3, and H3K27me3 were normalized to total H3. (B) H3.3S31ph, CHK1 phosphorylated at serine 296 (pCHK1), total CHK1, H3T3ph, H3S10ph, H3S28ph, H3.3, and total H3 were re-analyzed using histone-rich extracts of tibialis anterior muscle obtained in our previous study [[8](#_ENREF_8)]. (C) Correlation analysis between H3.3S31ph and both pCHK1 and total CHK1 was performed using all data points from the western blot results shown in panel B. H3.3 levels are presented as the ratio relative to H3.1/3.2 (panel A). H3.3S31ph levels were normalized to H3.3 (panel A). H3K4me3 (panel A), H3K27me3 (panel B), pCHK1 (panel B), total CHK1 (panel B), H3T3ph (panel B), H3S10ph (panel B), and H3S28ph (panel B) were normalized to total H3. Data are presented as mean ± SD (n=3 biological replicates per time point). Statistical significance was assessed by unpaired t-test (panels A and B) and Pearson correlation analysis (panel C). p-values are shown when < 0.05.

Figure 2: Effects of viral vector administration on the protein expression. HA tag, H3.3, H3.3S31ph, H3K4me3, H3K9me3, H3K27me3, and total H3 were analyzed by western blotting using histone-rich extracts from the tibialis anterior muscle. Representative blots and the quantitative data from the Stuffer, H3.3, and H3.3S31E groups (control mice from each group) are shown at 40 weeks of age (A), 50 weeks (B), and 60 weeks (C). The values were normalized with the respective total H3 levels. Viral vectors were intramuscularly injected at 30 weeks of age. Data are presented as mean ± SD (n=3 biological replicates per group). Statistical significance was assessed by one-way ANOVA followed by Scheffé’s post hoc test. p-values are shown when < 0.05.

Figure 3: Responses of gene expression to acute exercise. Twenty genes previously identified as being transcriptionally upregulated in response to acute exercise in mouse tibialis anterior muscle [[18](#_ENREF_18)] were analyzed in non-exercise controls (Cont) and 2 hours post-exercise (Exer) in the Stuffer, H3.3, and H3.3S31E groups at 40, 50, and 60 weeks of age. Gene expression was assessed as a gene set; dot plots represent values obtained from individual genes. Data were obtained from all biological replicates (n=3 per group) and averaged for each group. Expression values were normalized to their respective non-exercise control (set to 1). Data are presented as mean ± SD in dot plots. Statistical significance was assessed by one-way ANOVA followed by Scheffé’s post hoc test. p-values are shown when < 0.05.

Figure 4: Responses of histone and histone modification distribution to acute exercise. The distributions of HA tag (A), H3.3 (B), total H3 (C), H3K4me3 (D), H3K9me3 (E), and H3K27me3 (F) at the regions 1 kb upstream (upper panels) and 1 kb downstream (lower panels) of TSS in the target loci were analyzed by ChIP-qPCR in non-exercise control (Cont) and 2 hours post-exercise (Exer) conditions in the Stuffer, H3.3, and H3.3S31E groups at 60 weeks of age. Distribution of each target protein was assessed as a gene set; dot plots represent values obtained from individual genes. Chromatin from all biological replicates (n = 3 per group) was pooled for the ChIP reaction. The ChIP assay was performed twice to confirm data reproducibility. % input values were normalized to an average value of 1 for each gene. Data are presented as mean ± SD in dot plots. Statistical significance was assessed by one-way ANOVA followed by Scheffé’s post hoc test. p-values are shown when < 0.05.

Figure 5: Indicators of histone turnover. The HA tag/H3.3 ratio (A) and H3.3/total H3 ratio (B) were calculated using % input values shown in Figure 4. Each ratio was further normalized to an average value of 1 for each gene. Data are presented as mean ± SD in dot plots. Statistical significance was assessed by one-way ANOVA followed by Scheffé’s post hoc test. p-values are shown when < 0.05. Note: p-values for comparisons of the HA tag/H3.3 ratio to the Stuffer group are not displayed in the figure, as all corresponding groups in both the H3.3 and H3.3S31E conditions showed significant differences from the Stuffer group (p < 0.05).

Figure 1


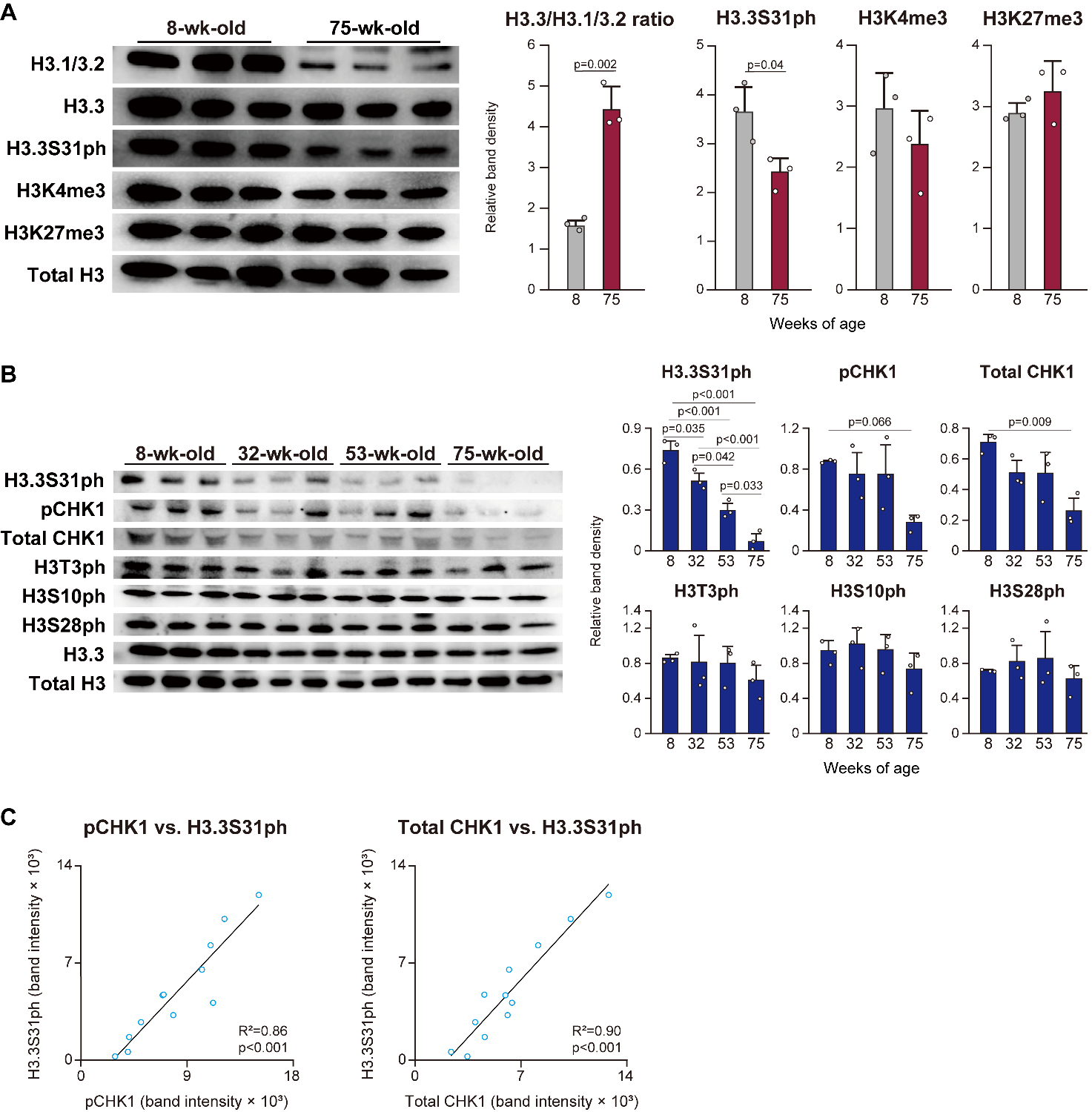


Figure 2


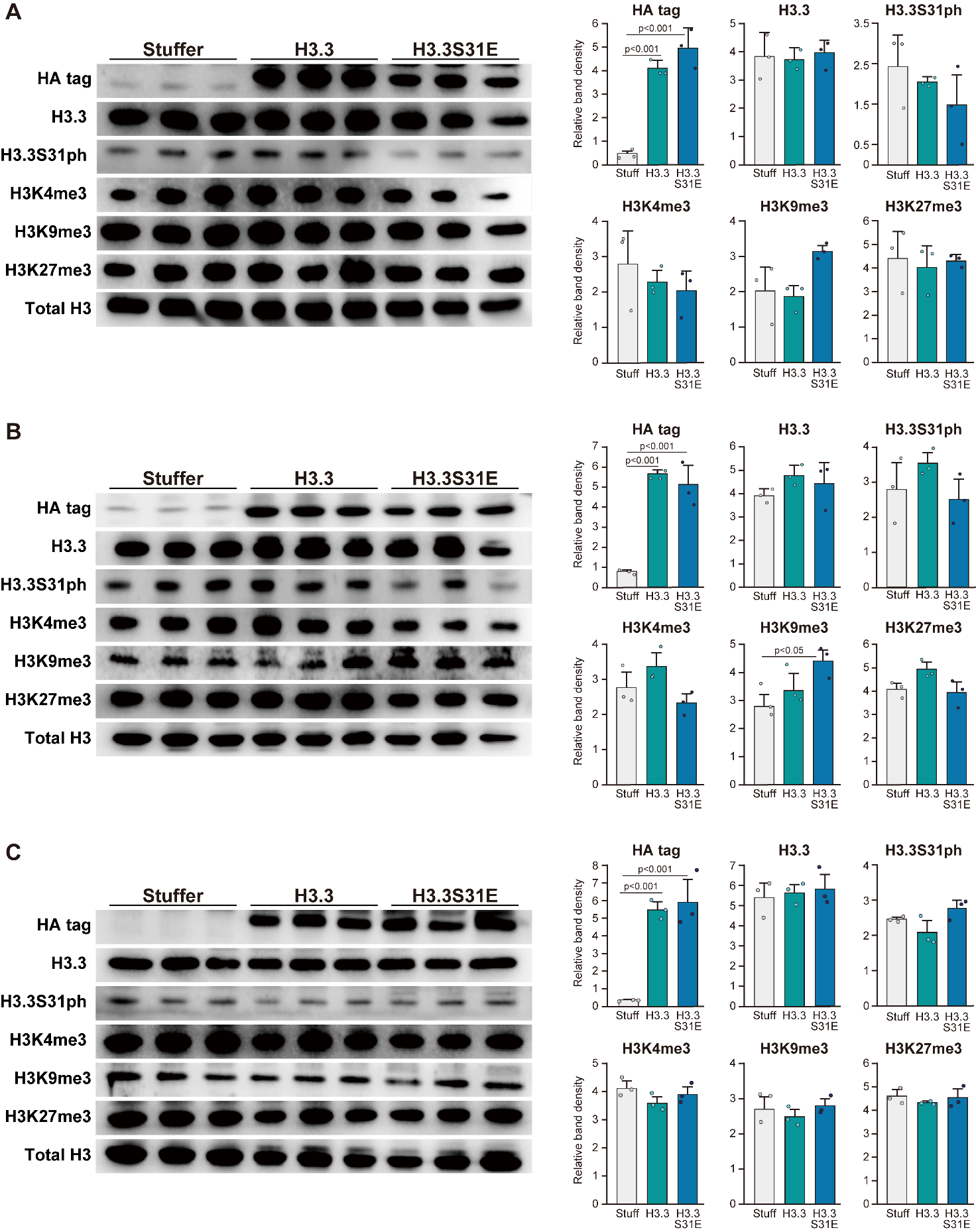


Figure 3


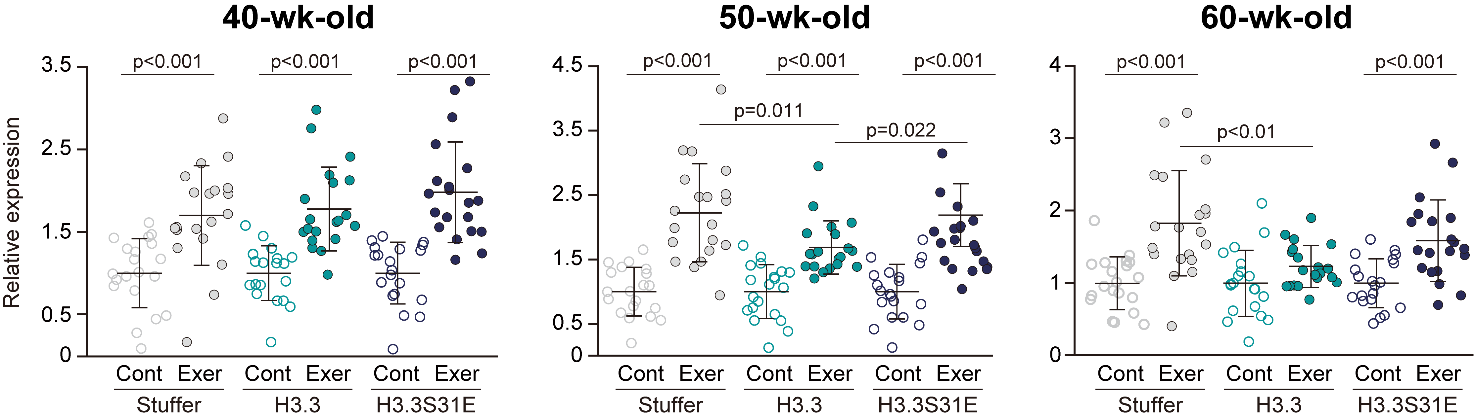


Figure 4


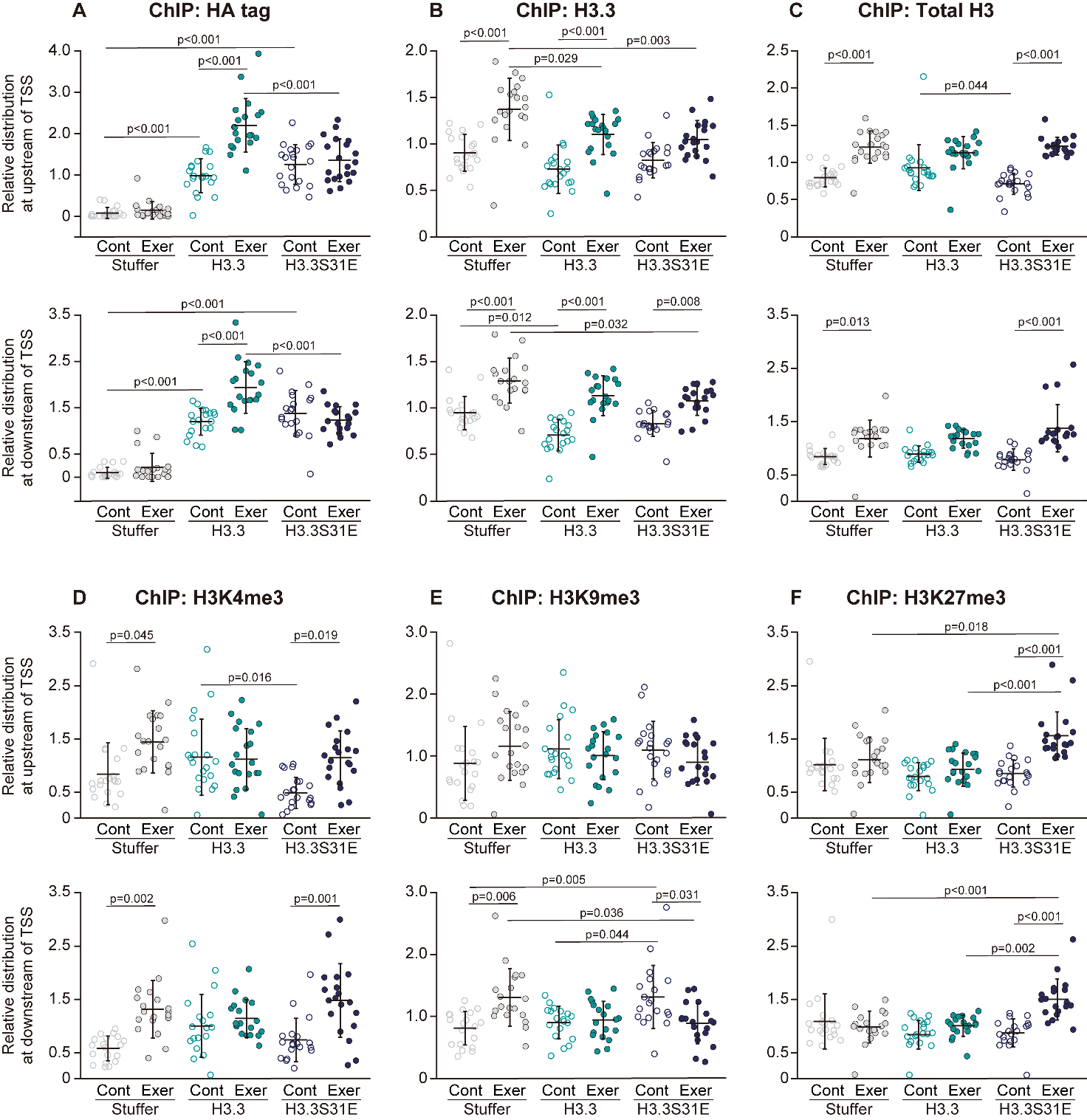


Figure 5


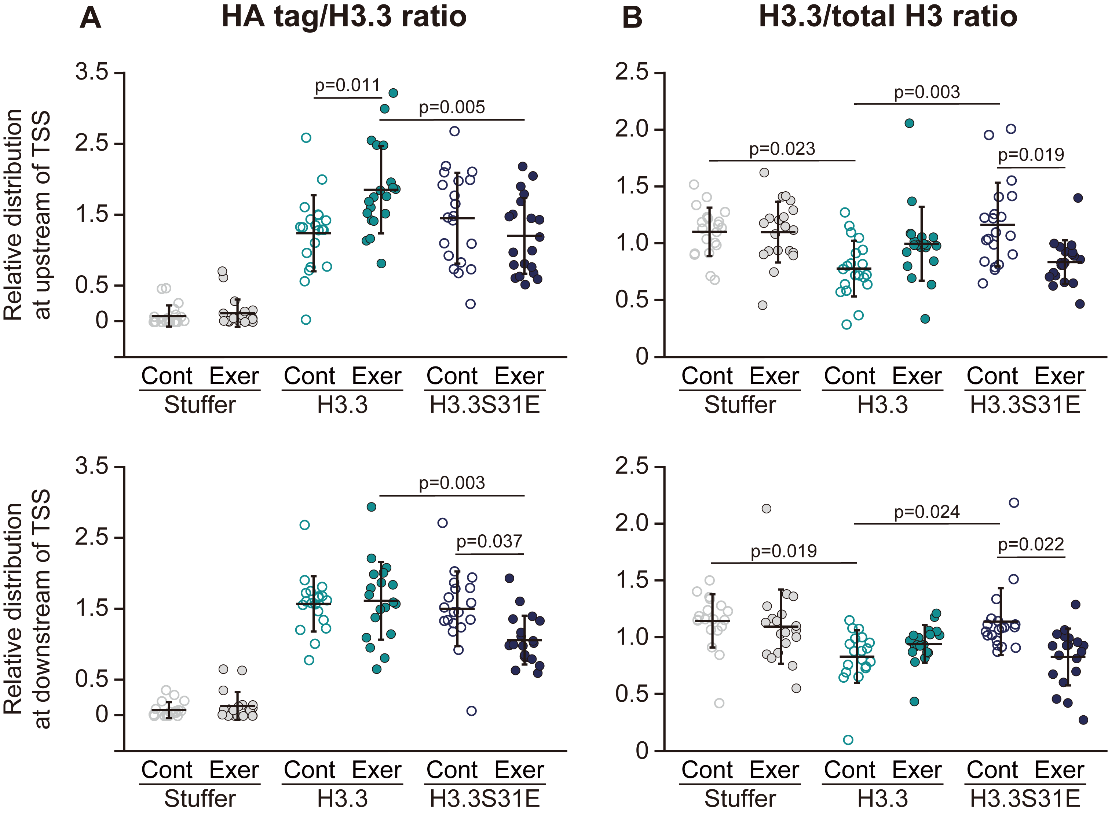

Supplement: Supplementary file 1 — Supplementary material 1. [file 42826_2025_254_MOESM1_ESM.docx]
