# Supplementary material for: Phosphorylation-mimicking histone H3.3 rescues exercise-induced gene responses in an epigenetic aging model of mouse skeletal muscle
Source: Lab Anim Res. 2025 Sep 24;41:25. doi: 10.1186/s42826-025-00254-6 (PMC12459025; doi:10.1186/s42826-025-00254-6)

Figure 1 H3.1/3.2


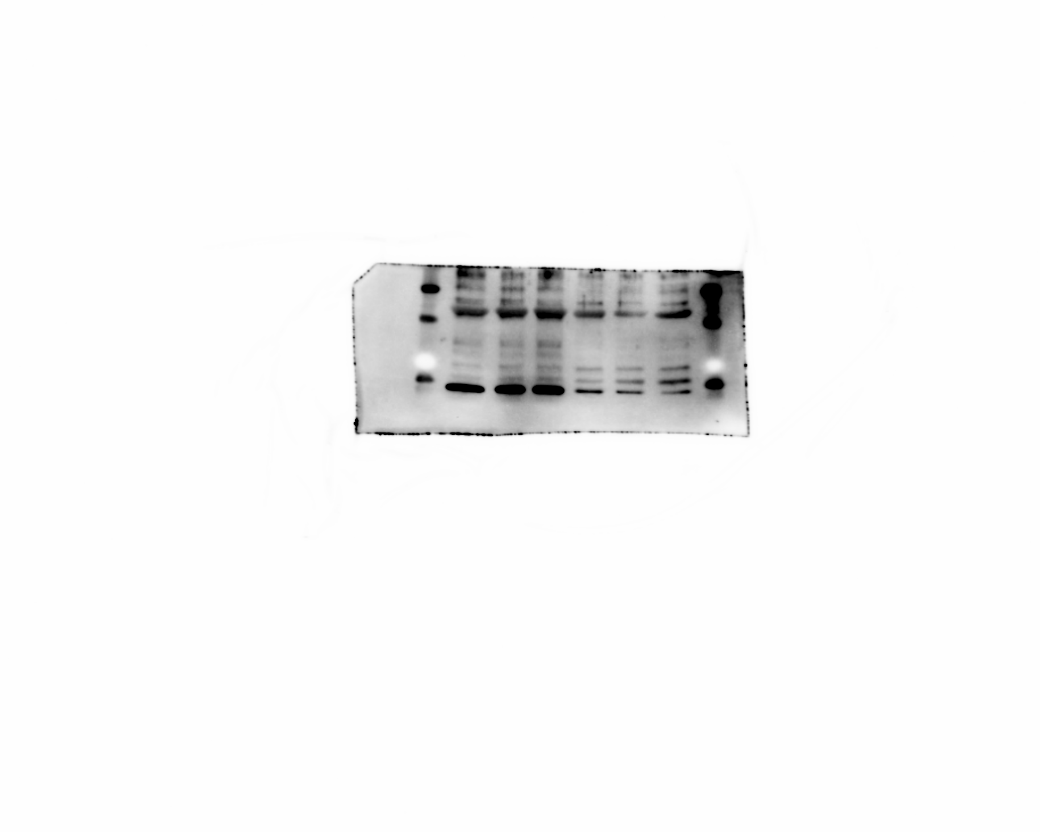


Figure 1 H3.3


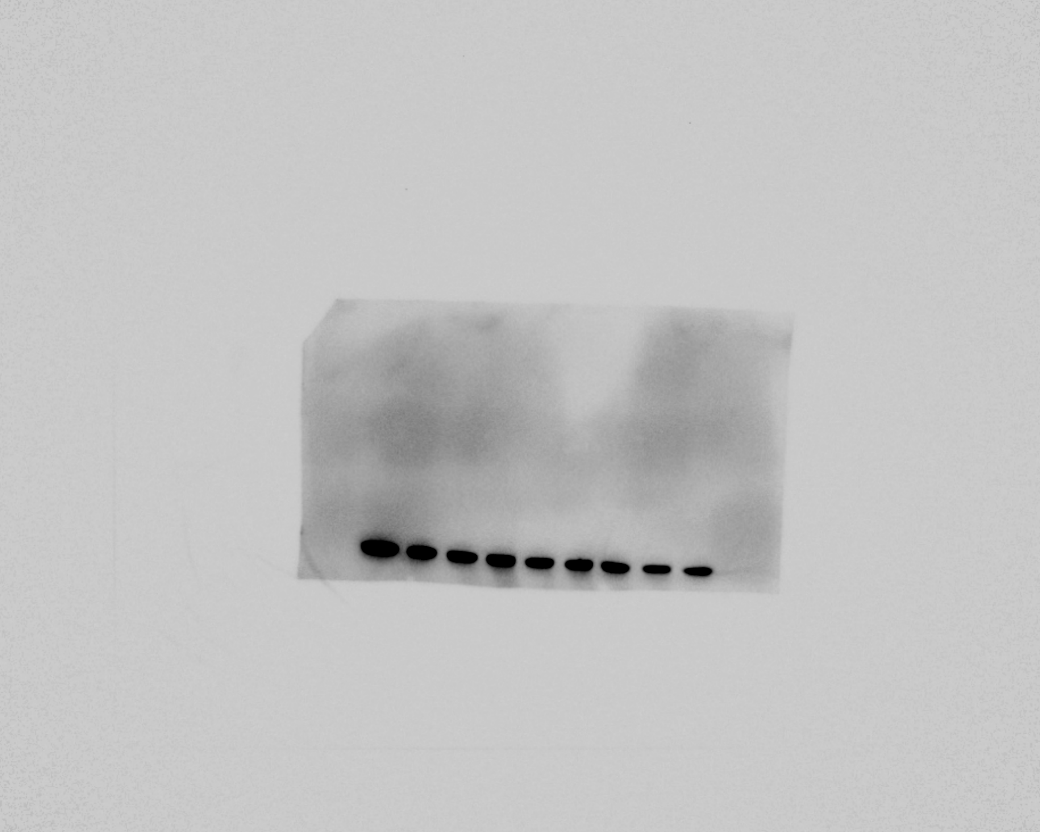


＊lanes 1-3: 8wk-TA, lanes 4-6: 75wk-TA, lanes 7-9: 8wk-soleus

Figure 1 H3.3S31ph


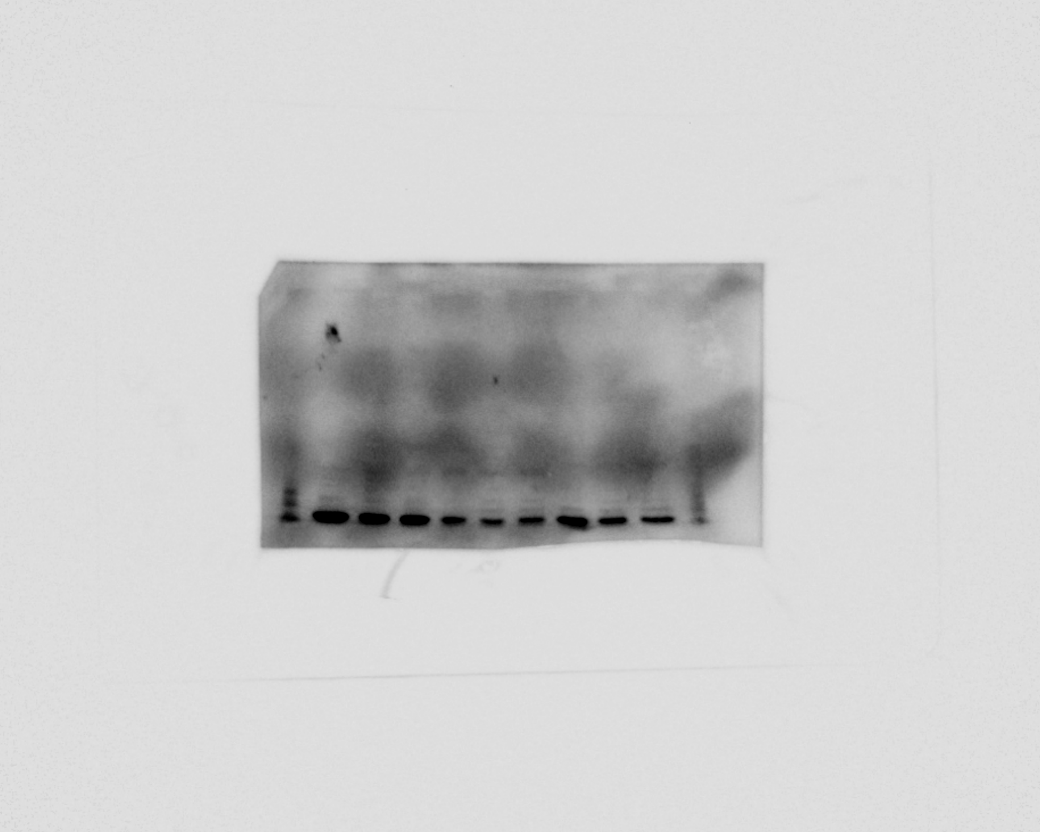


＊lanes 1-3: 8wk-TA, lanes 4-6: 75wk-TA, lanes 7-9: 8wk-soleus

Figure 1 H3K4me3


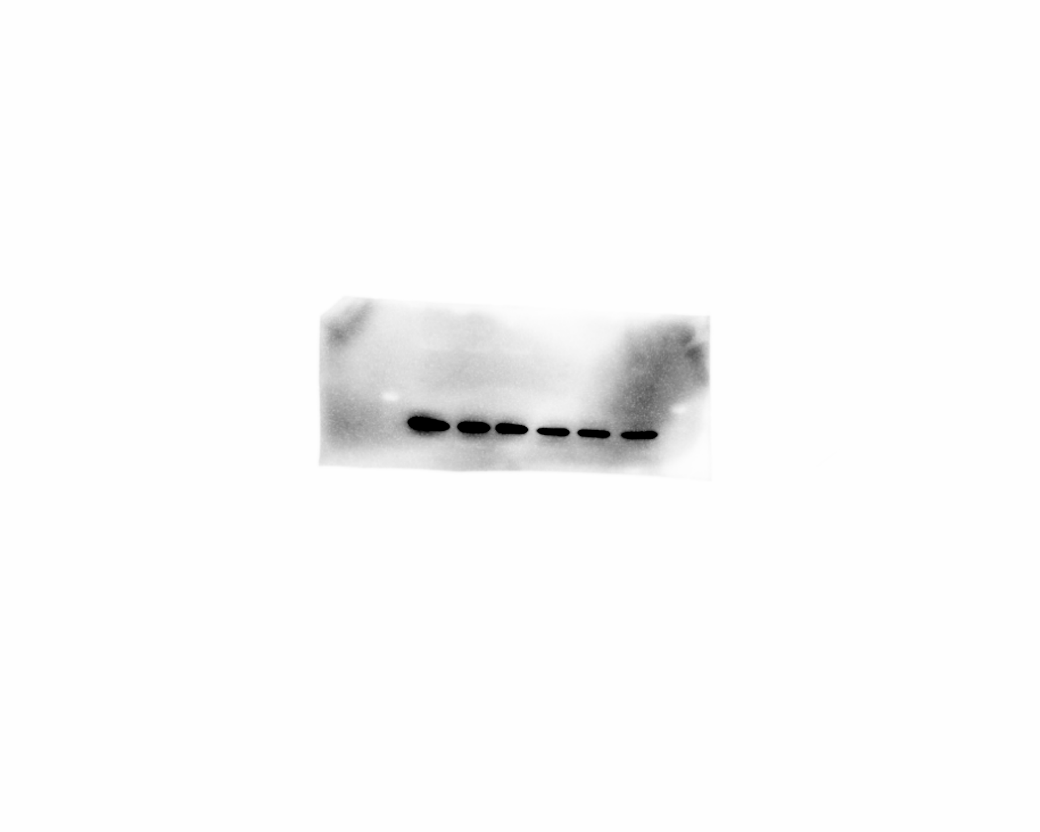


Figure 1 H3K27me3


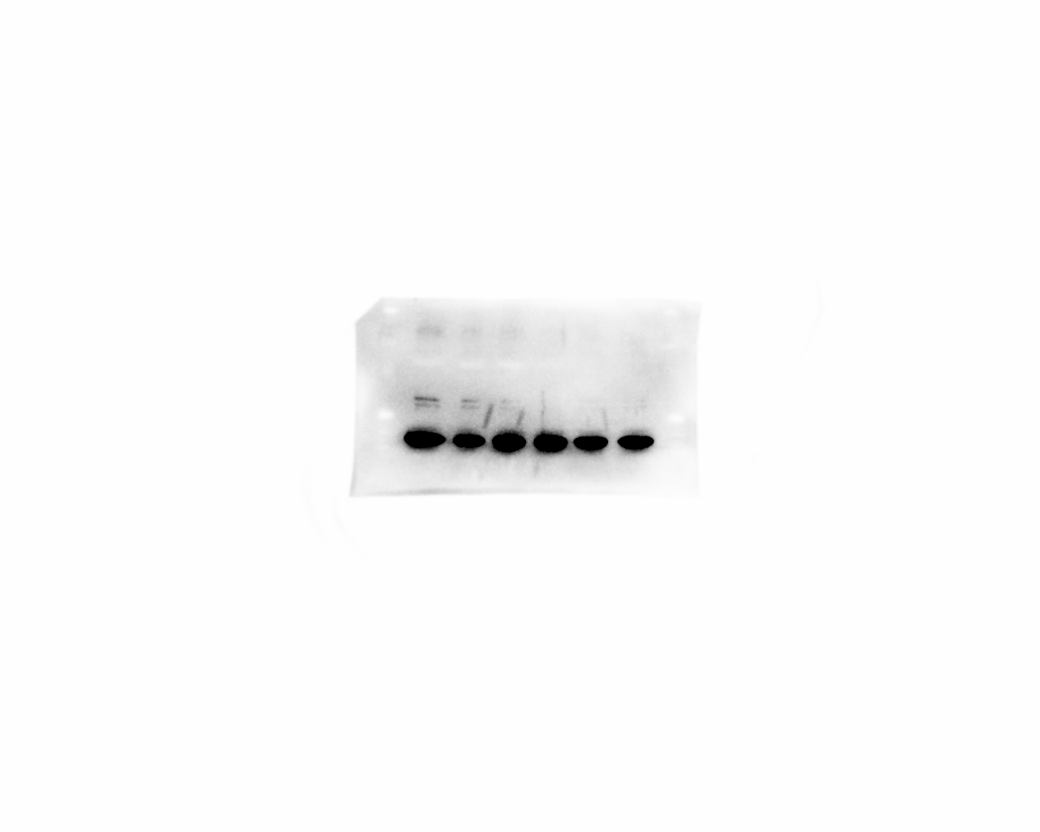


Figure 1 Total H3


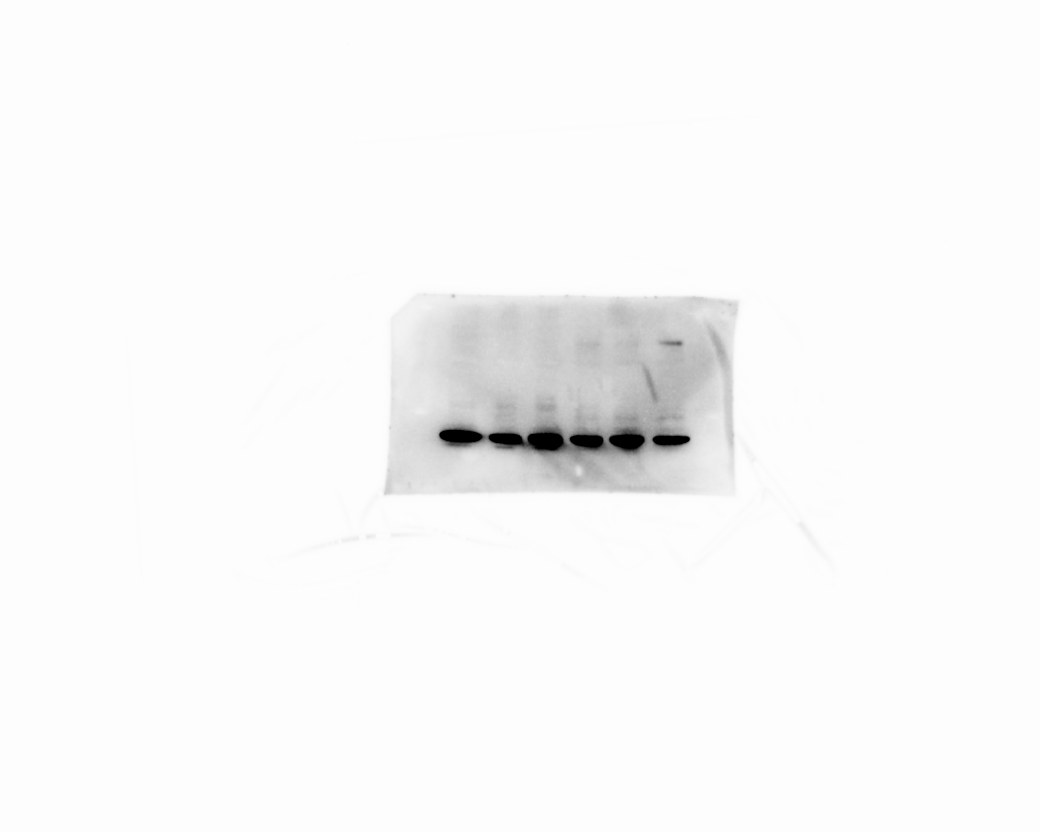


・Figure 1B H3.3S31ph


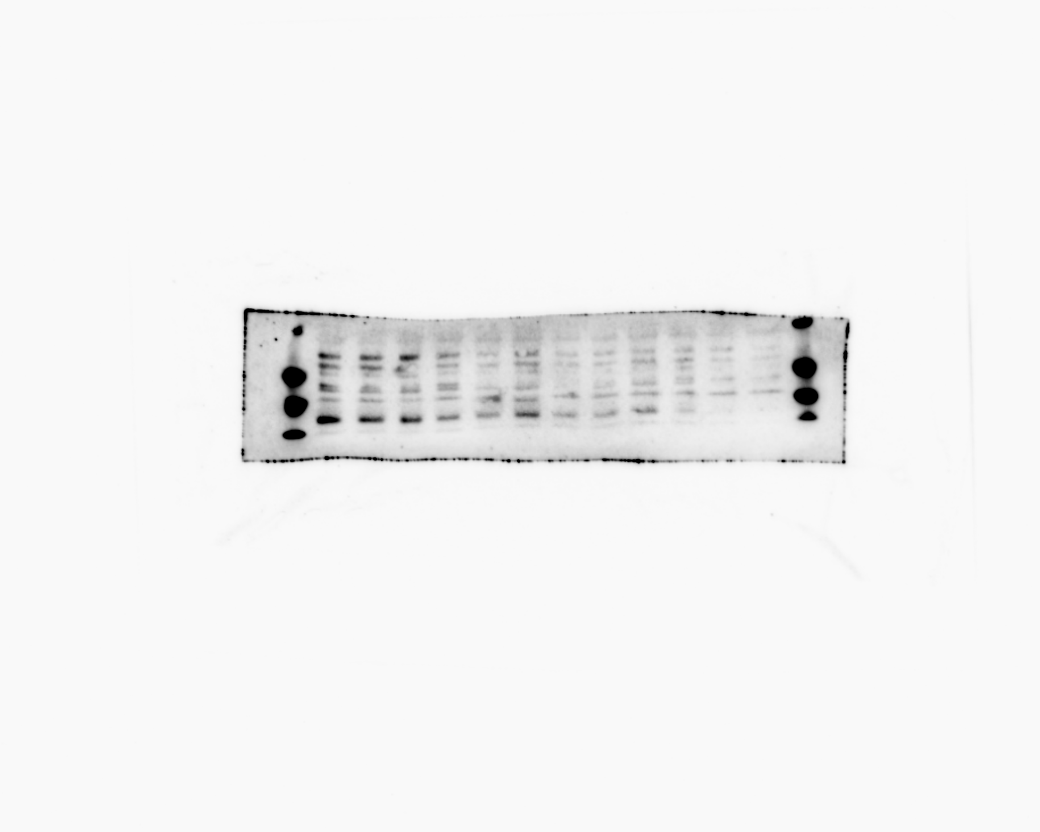


・Figure 1B pCHK1


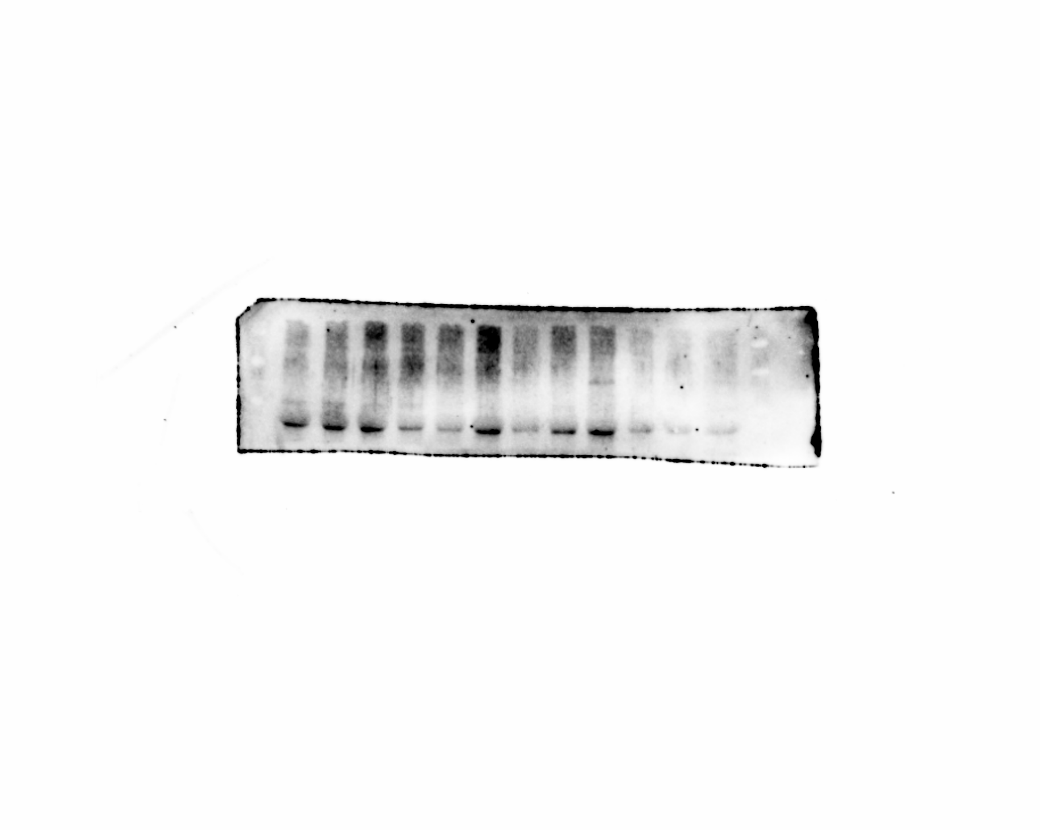


・Figure 1B Total CHK1


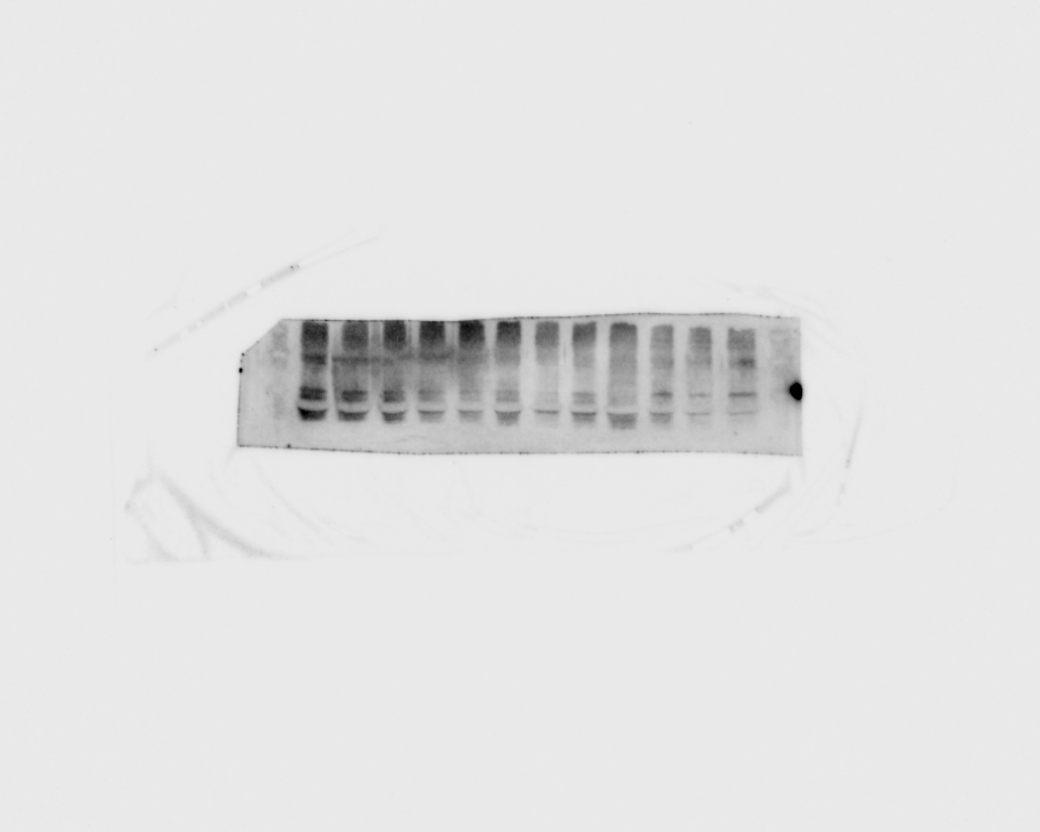


・Figure 1B H3T3ph


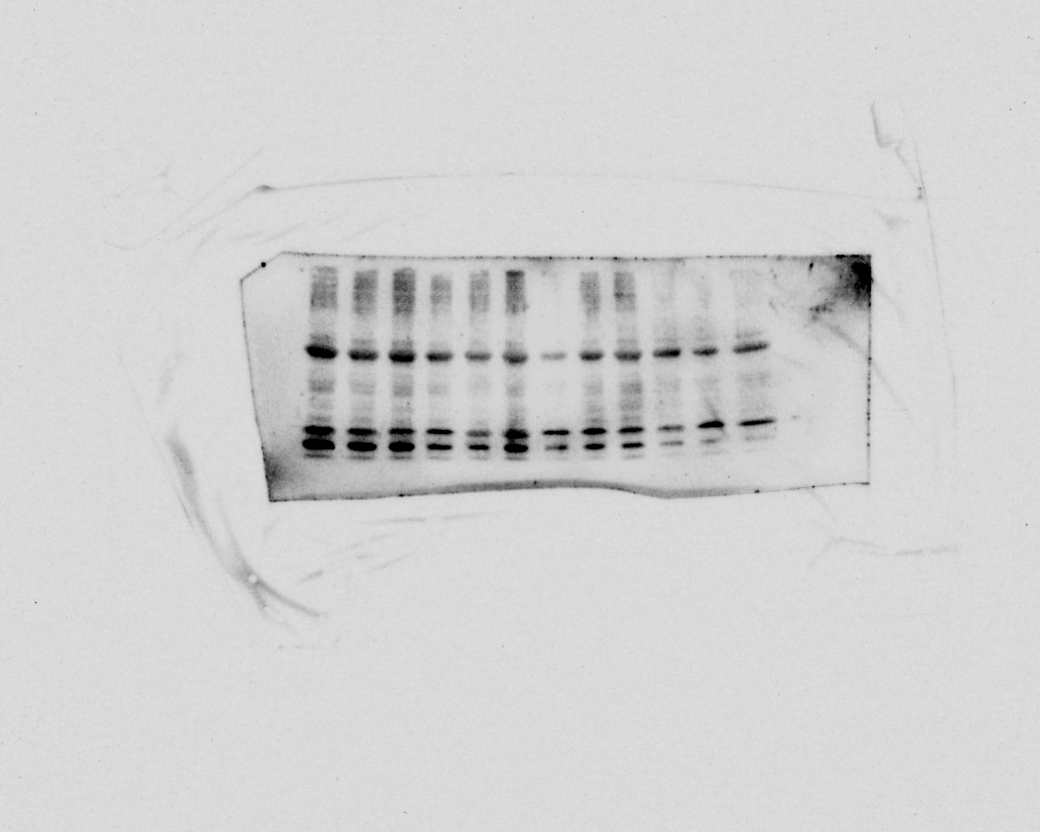


・Figure 1B H3S10ph


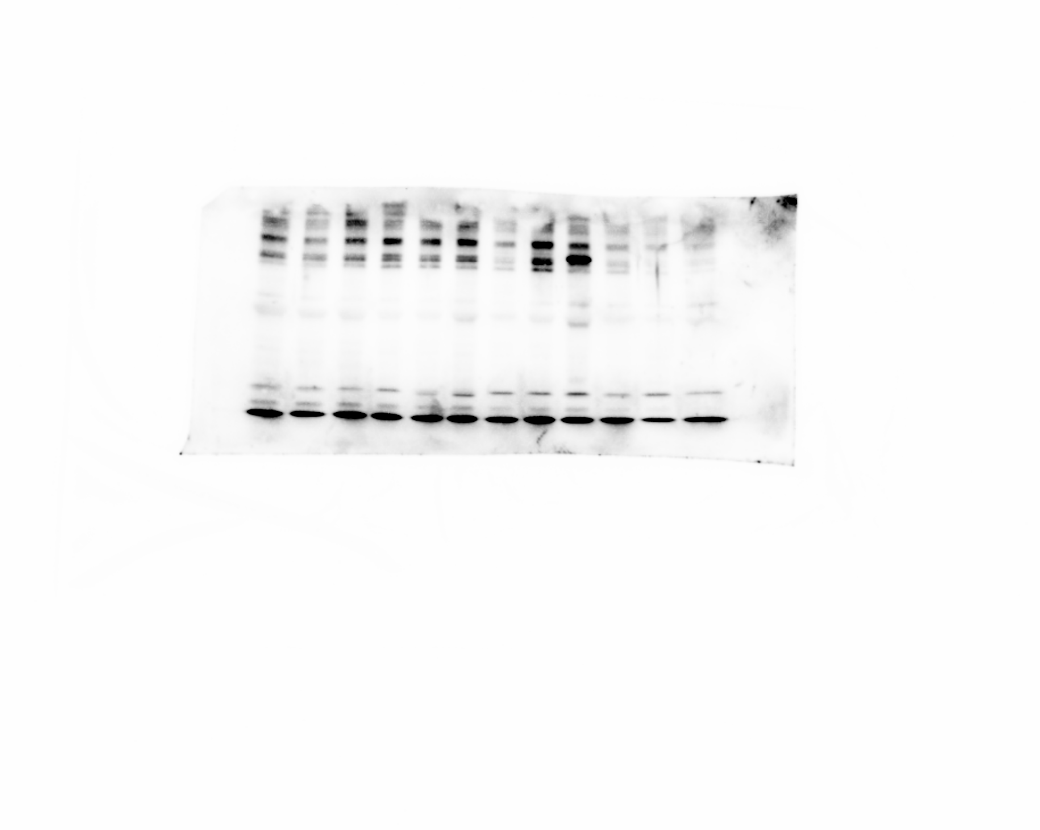


・Figure 1B H3S28ph


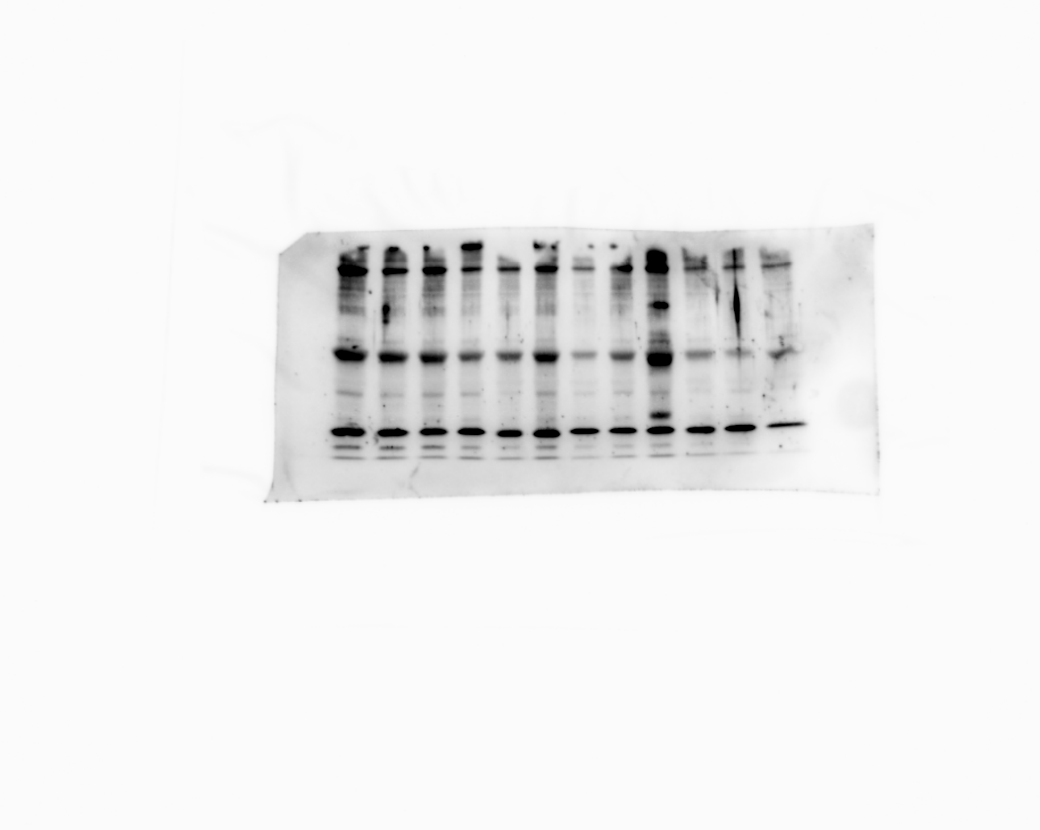


・Figure 1B H3.3


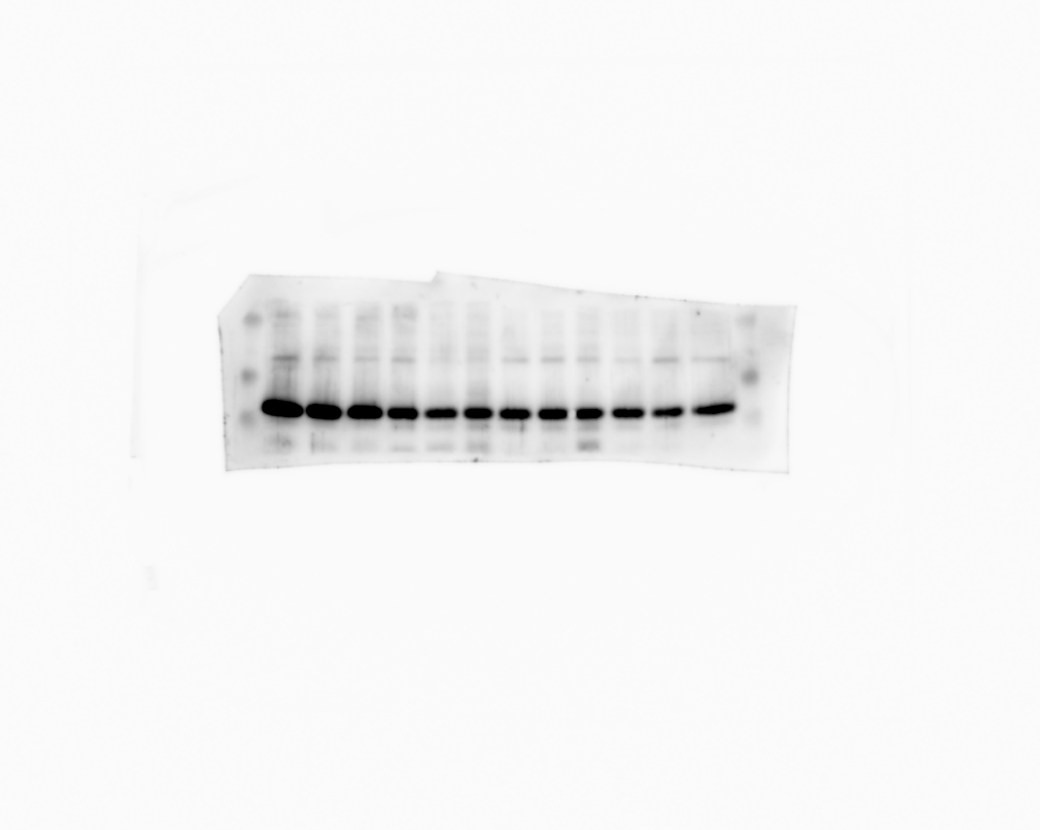


・Figure 1B Total H3


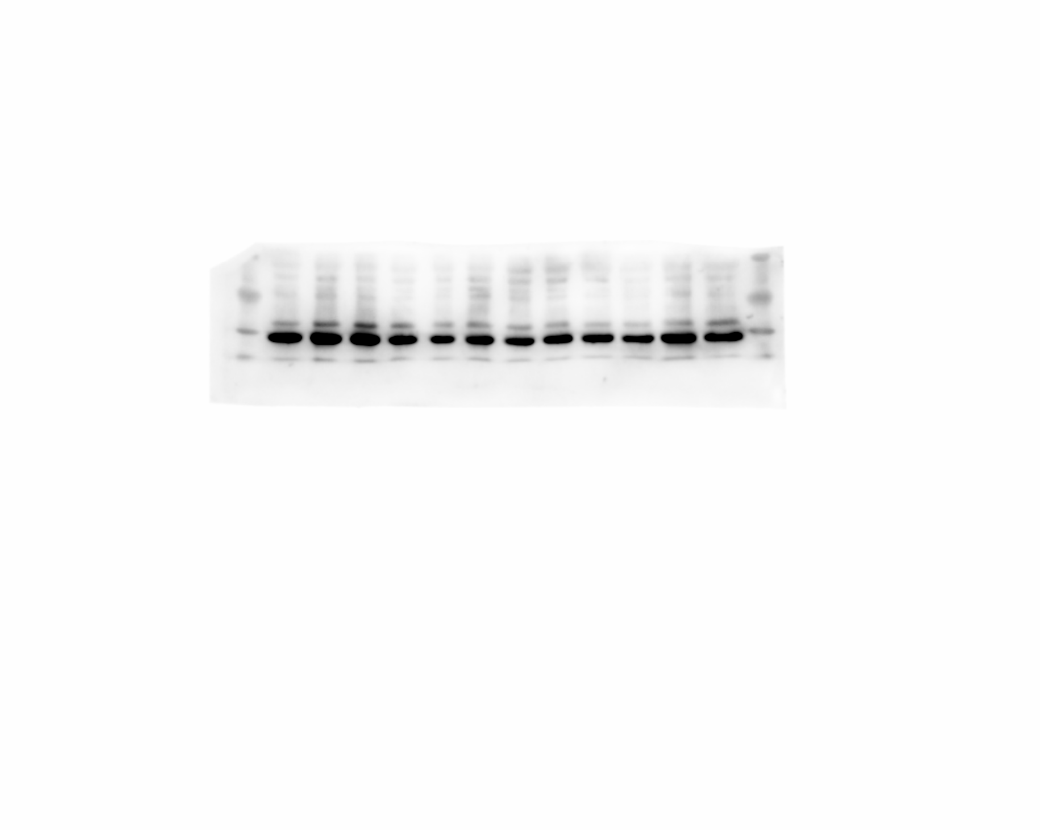


Figure 2A HA tag


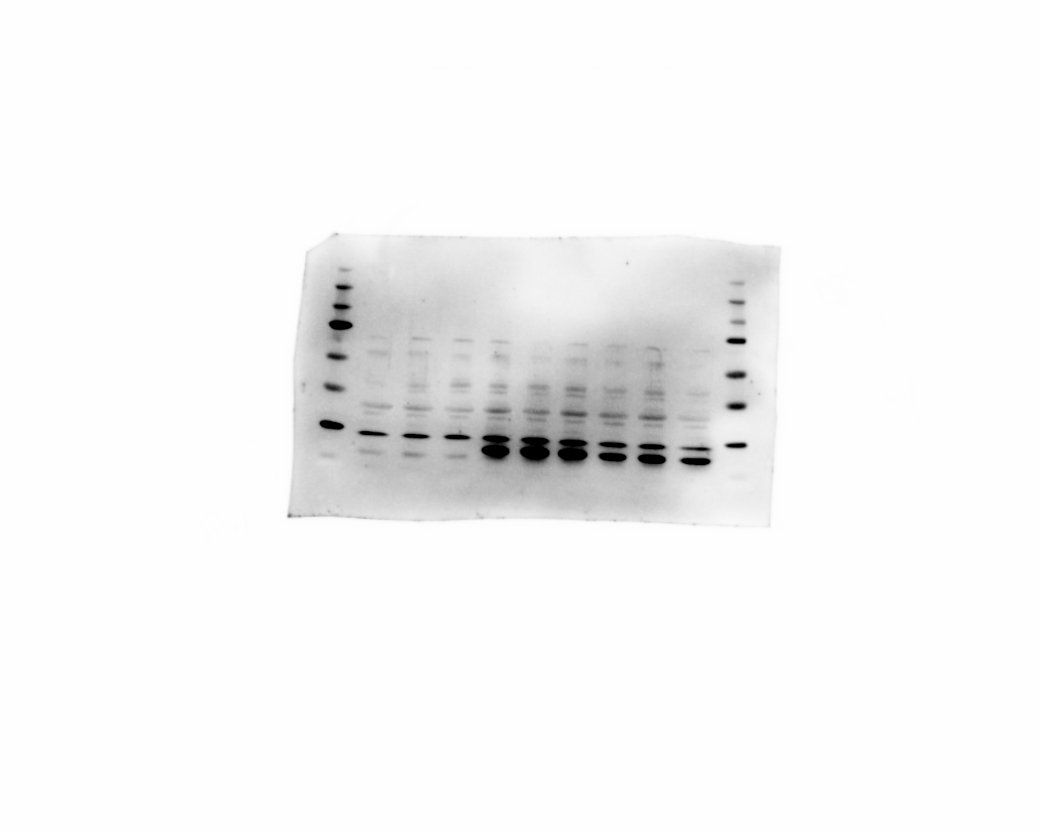


Figure 2A H3.3


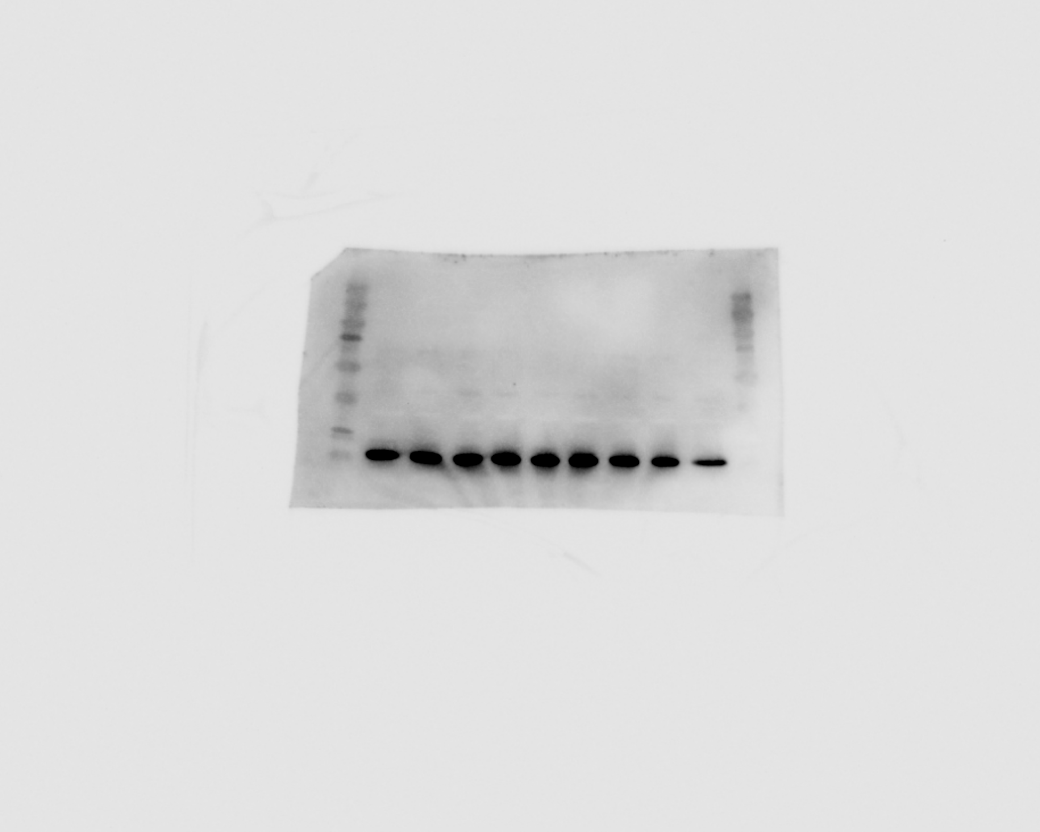


Figure 2A H3.3S31ph


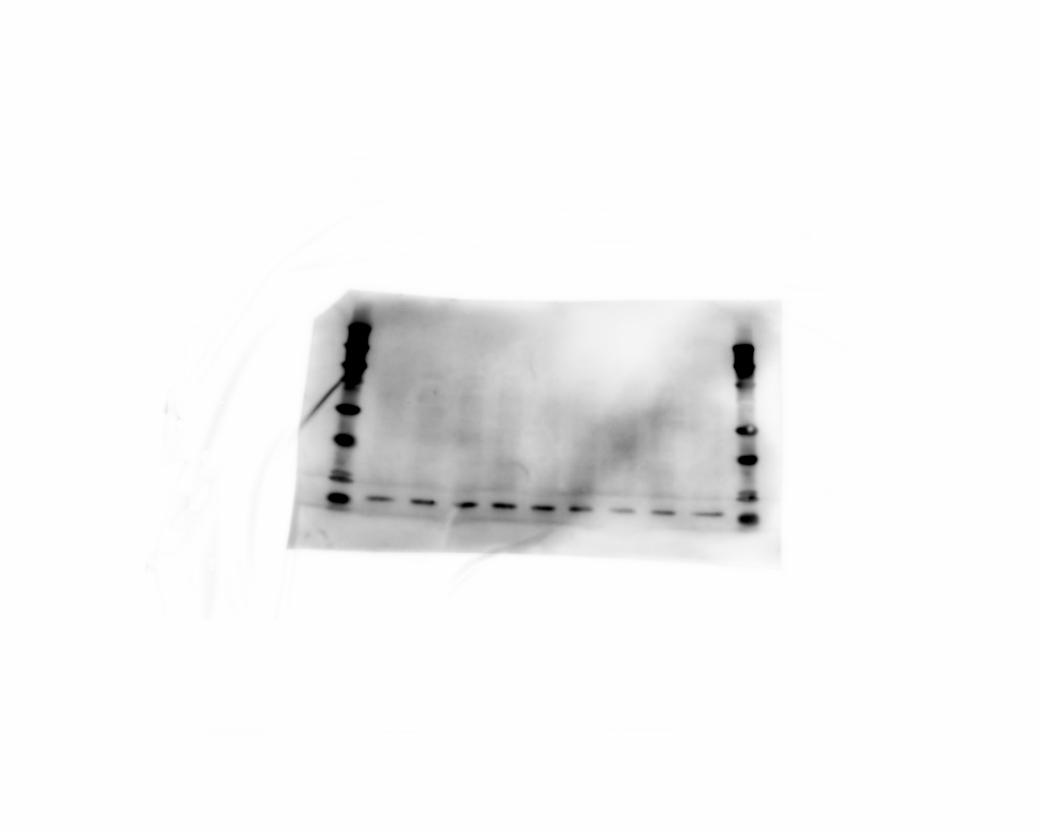


Figure 2A H3K4me3


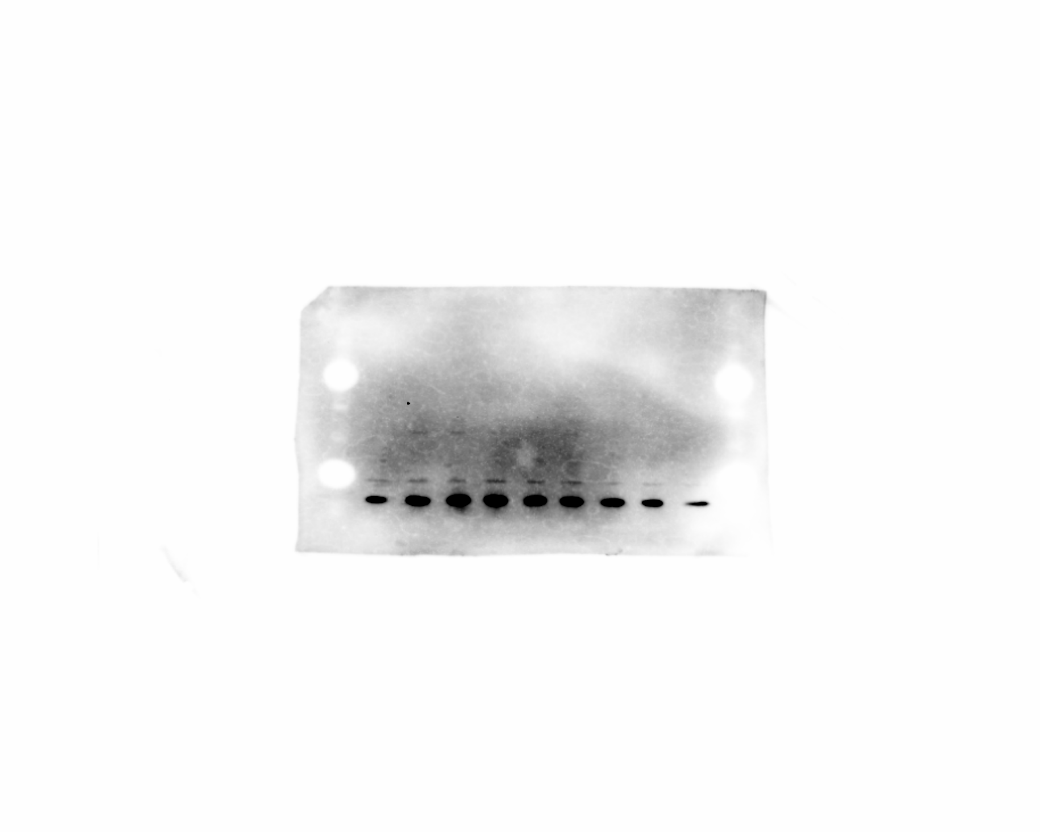


Figure 2A H3K9me3


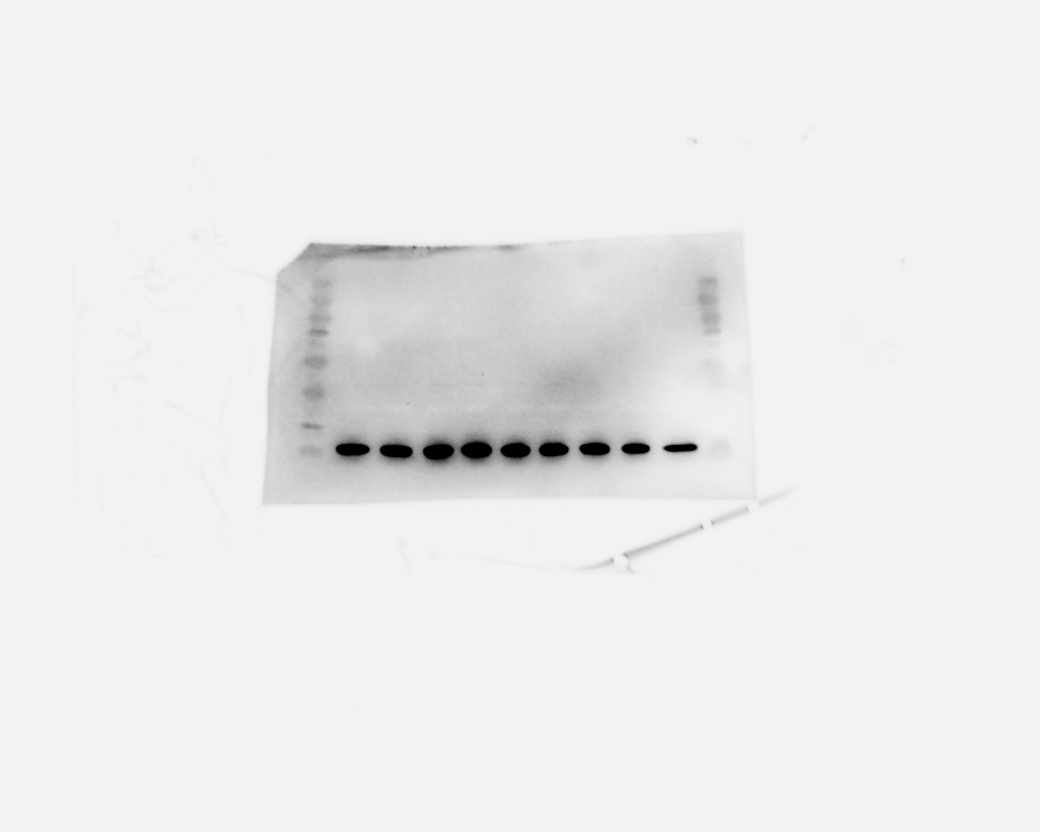


Figure 2A H3K27me3


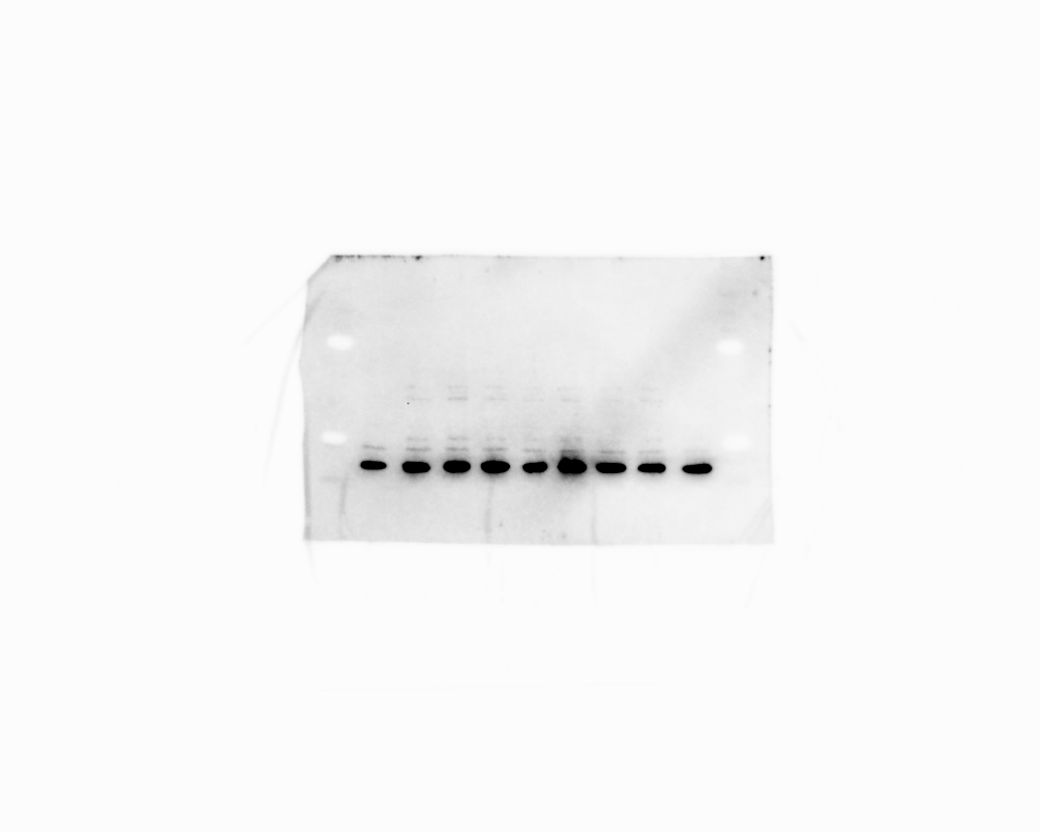


Figure 2A Total H3


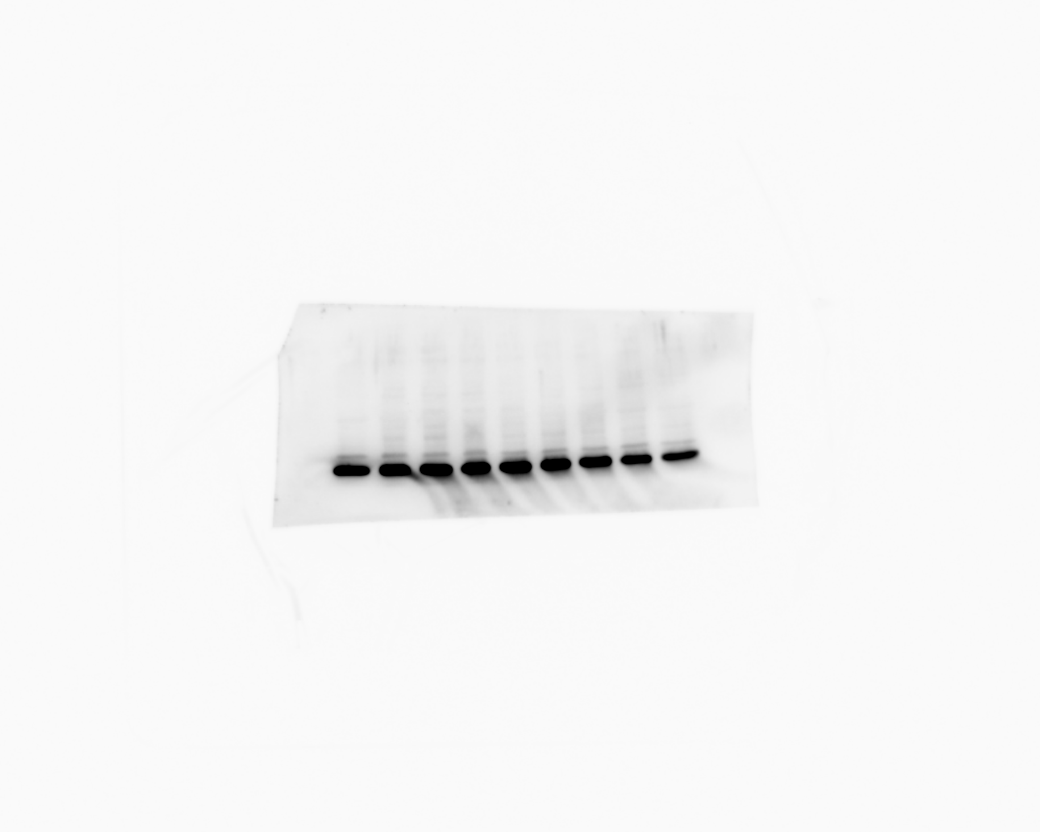


Figure 2B HA tag


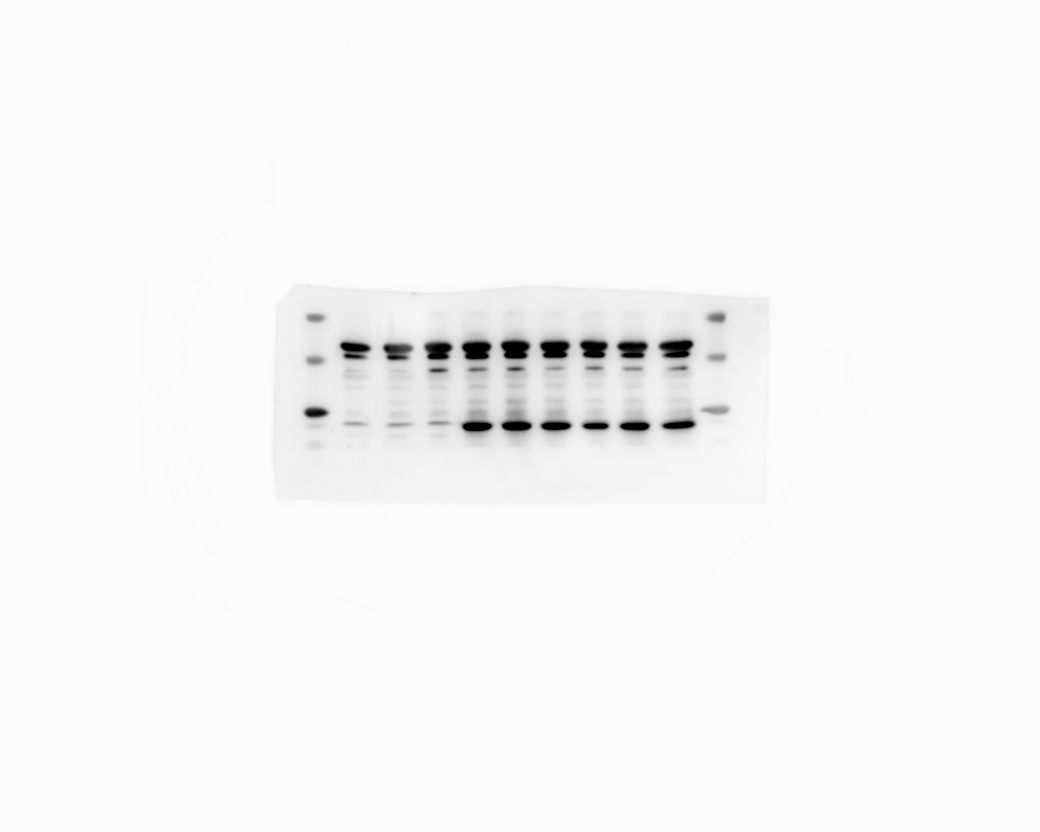


Figure 2B H3.3


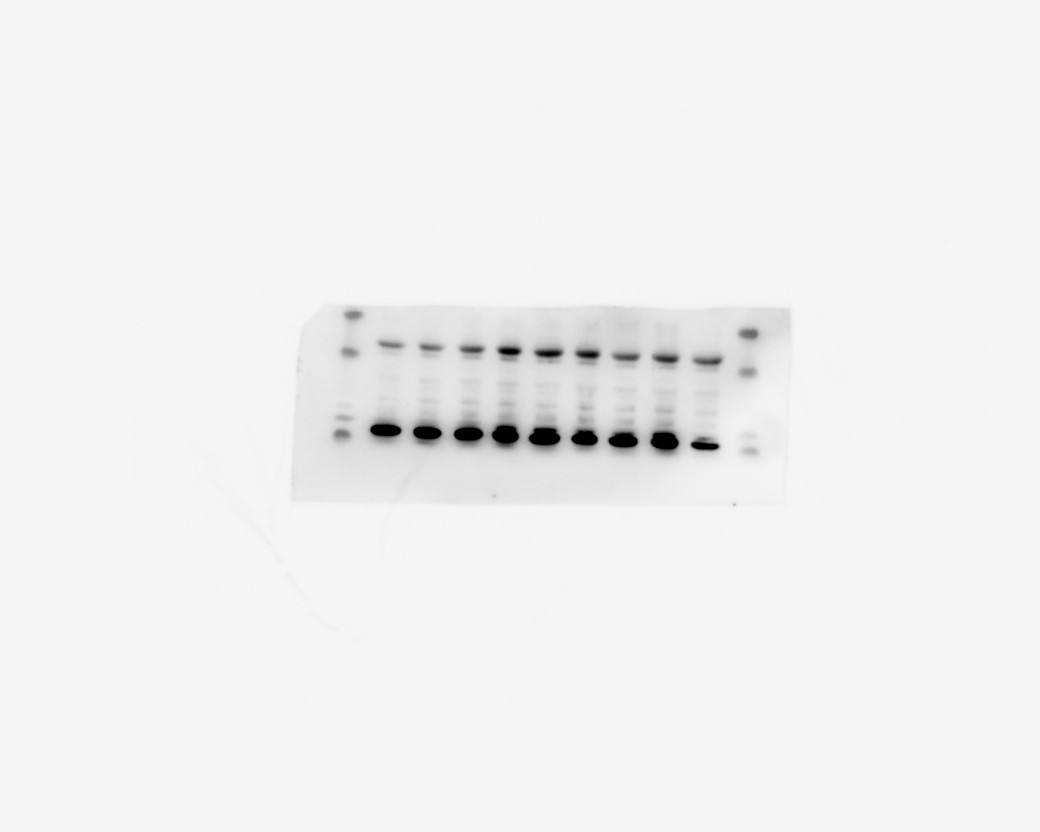


Figure 2B H3.3S31ph


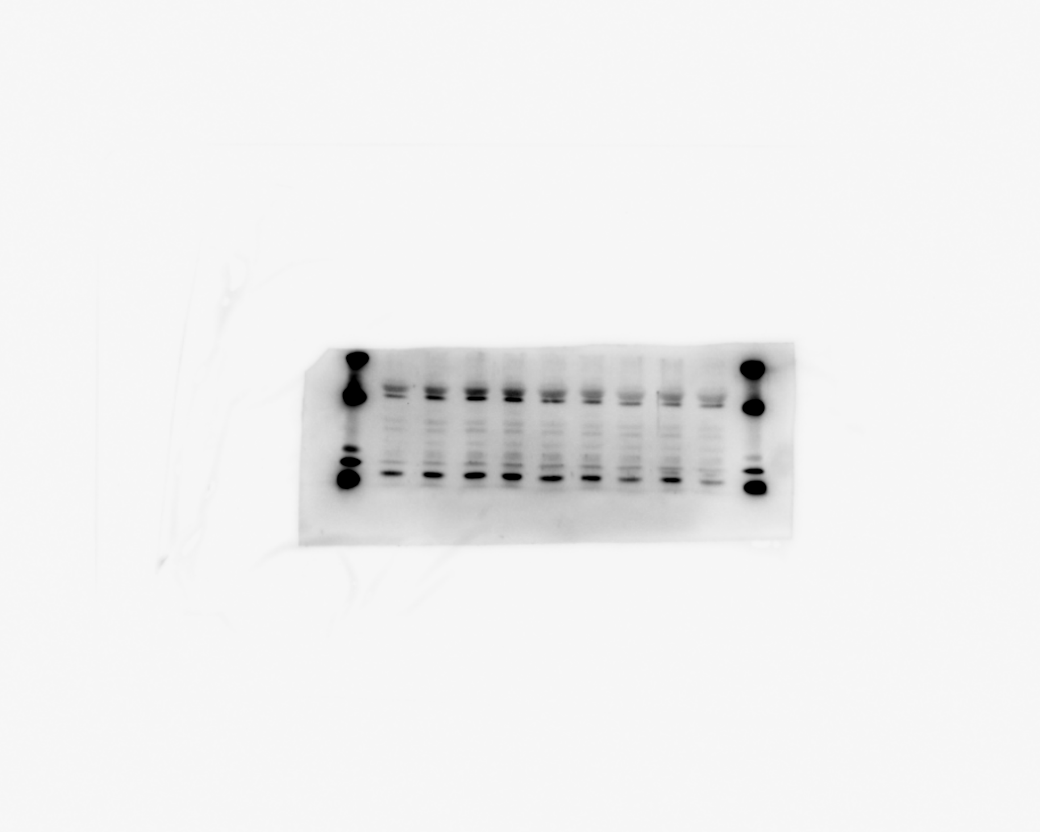


Figure 2B H3K4me3


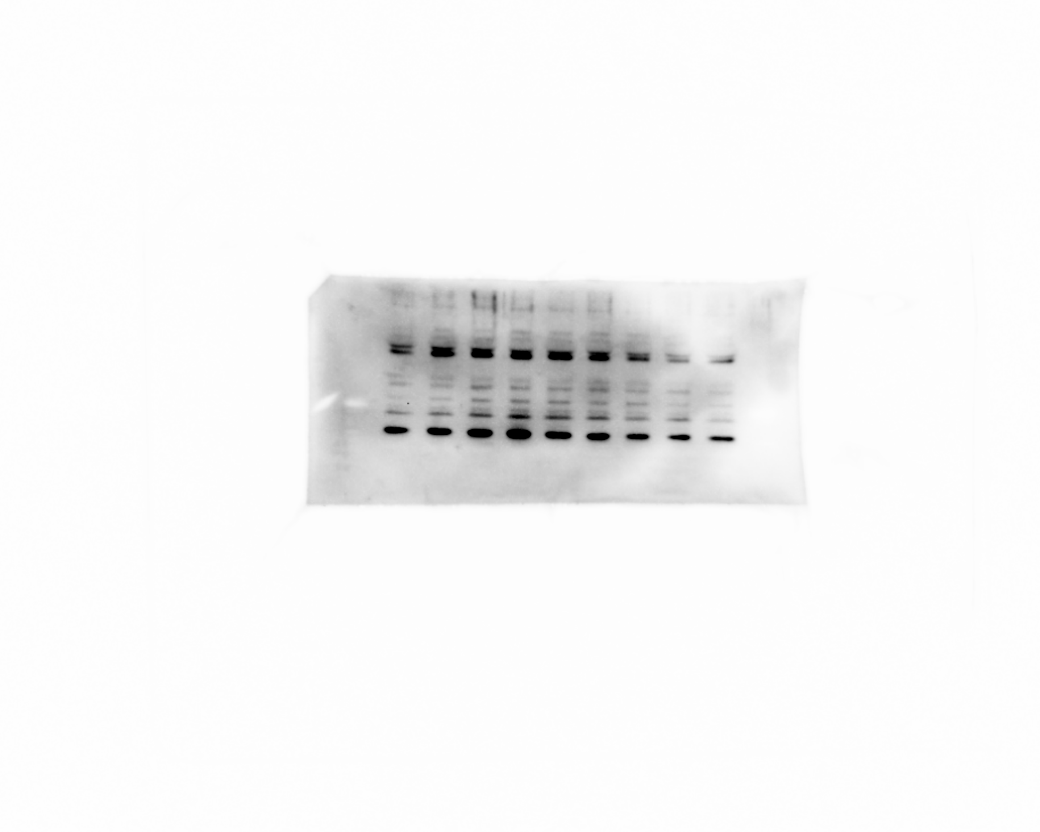


Figure 2B H3K9me3


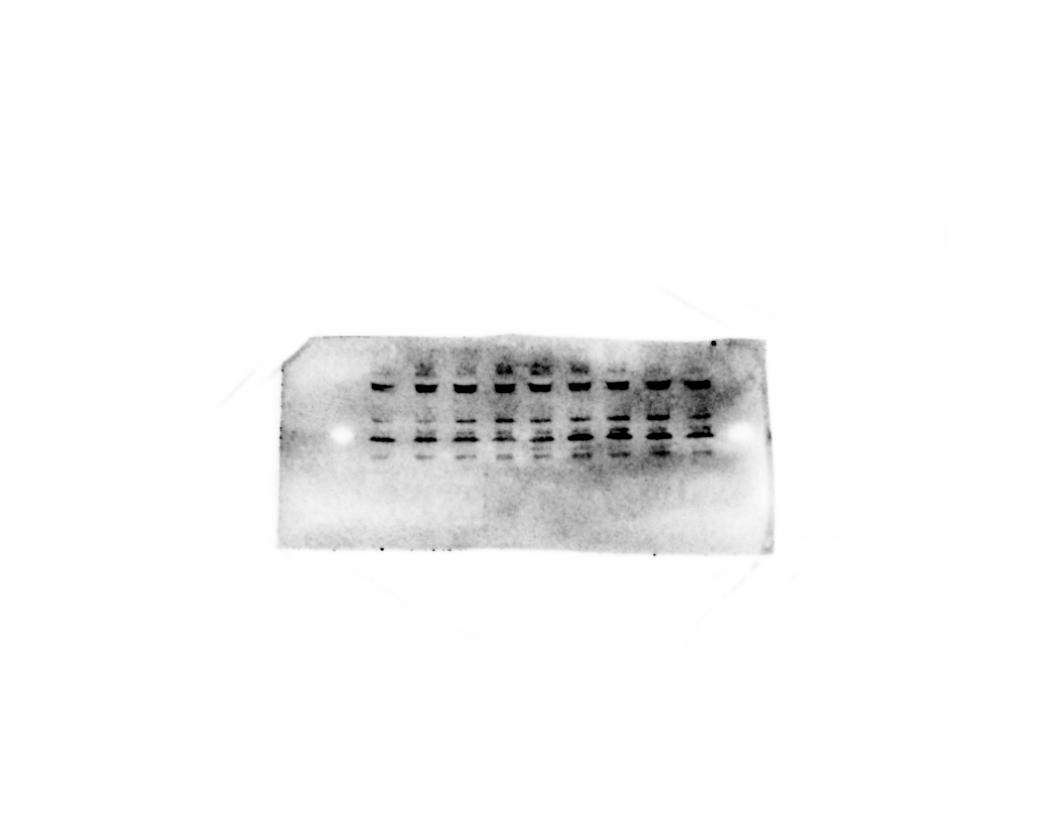


Figure 2B H3K27me3


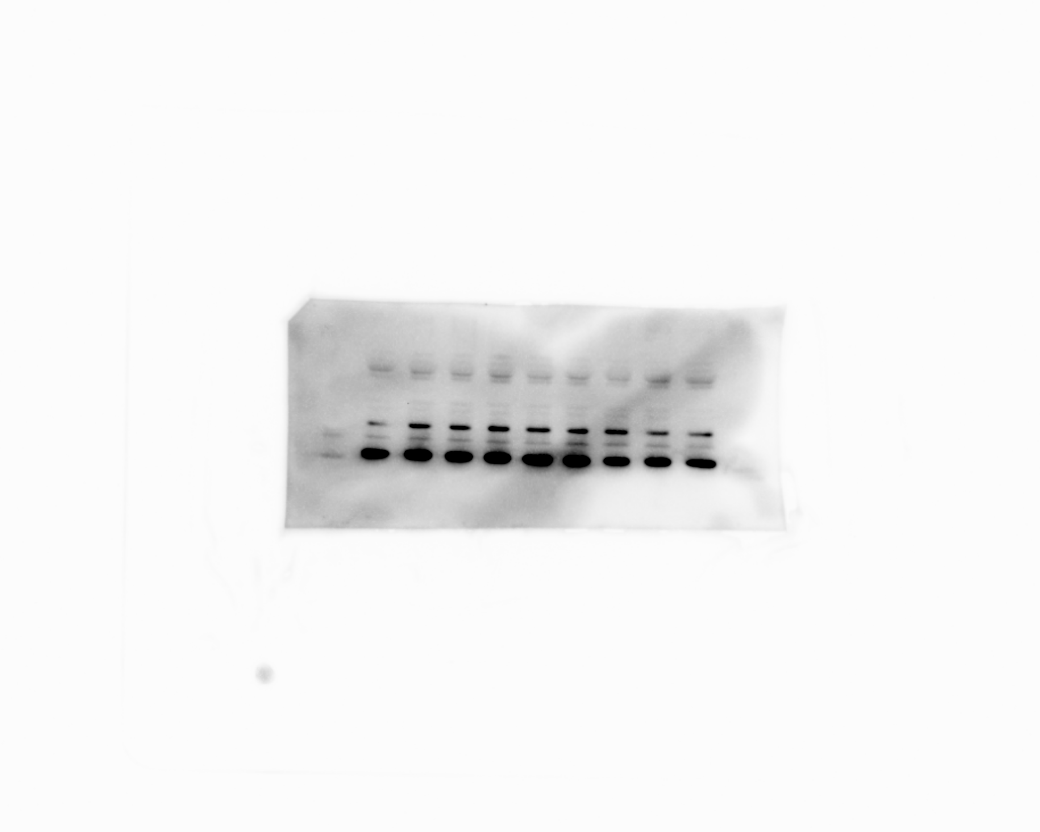


Figure 2B Total H3


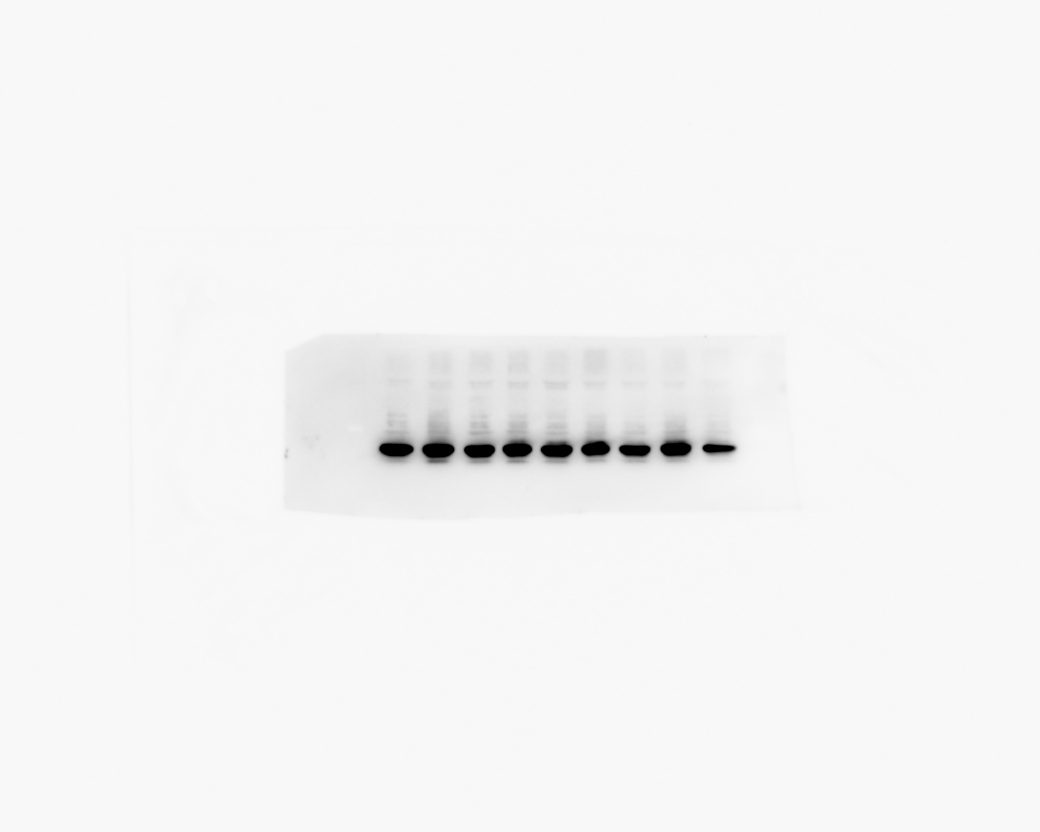


Figure 2C HA tag


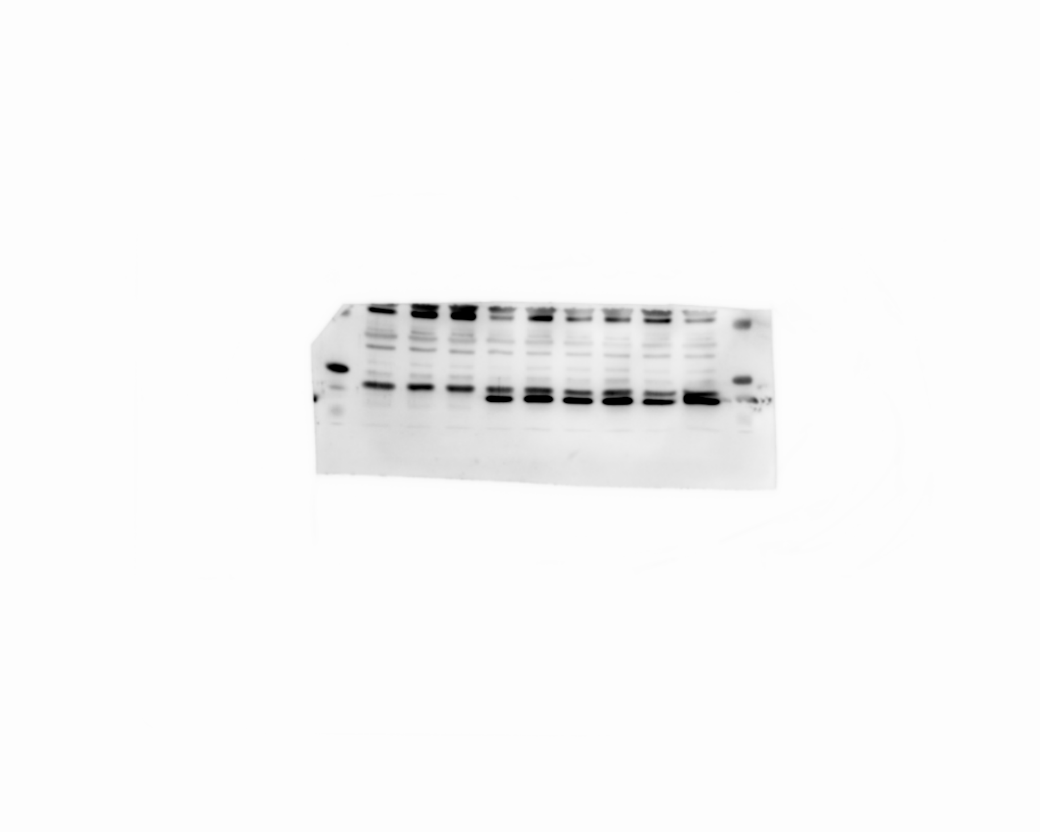


Figure 2C H3.3


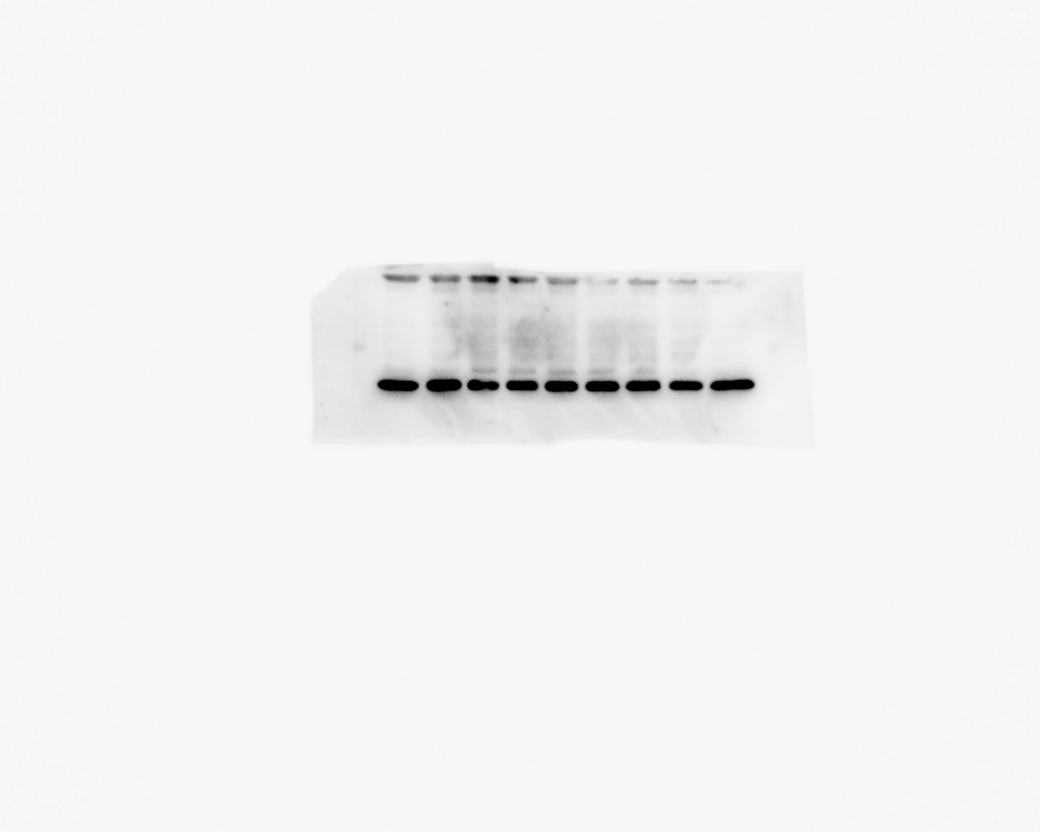


Figure 2C H3.3S31ph


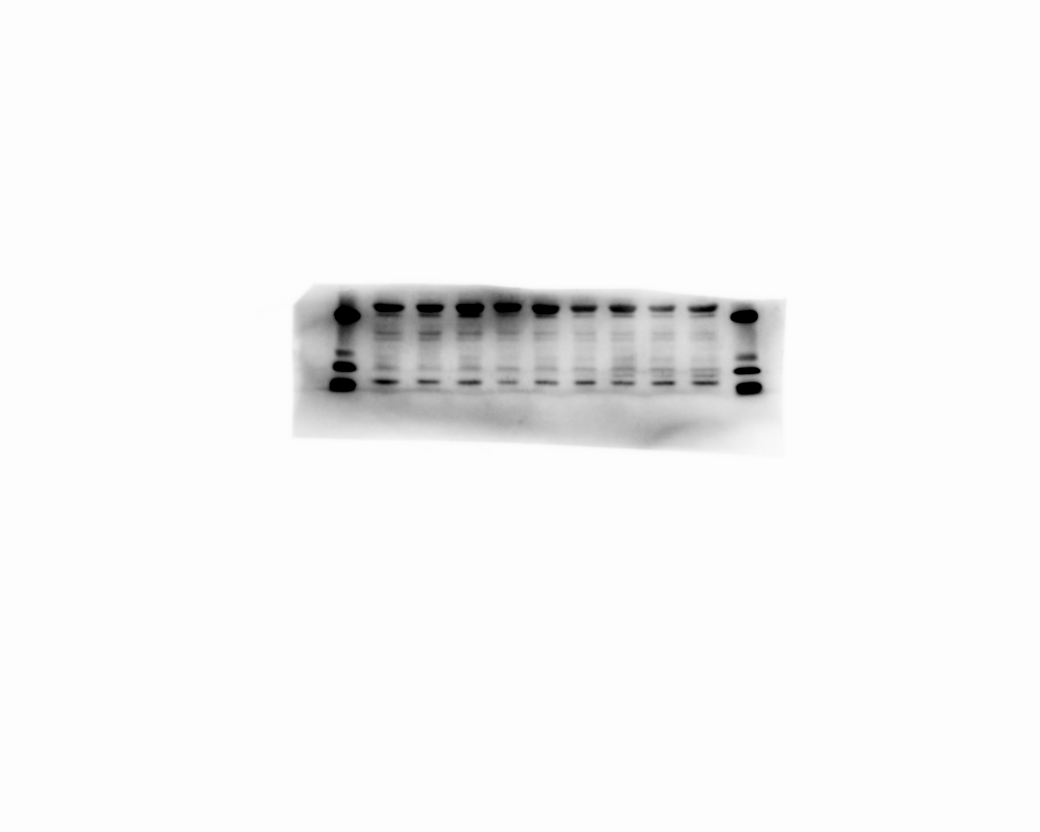


Figure 2C H3K4me3


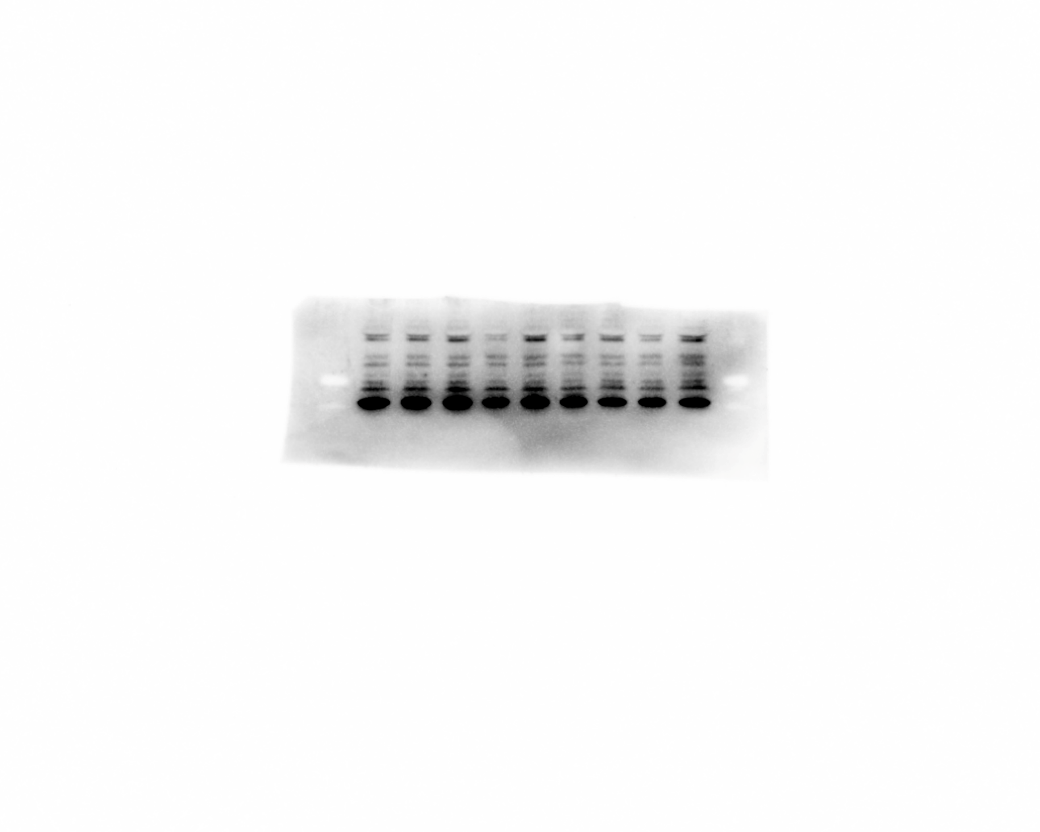


Figure 2C H3K9me3


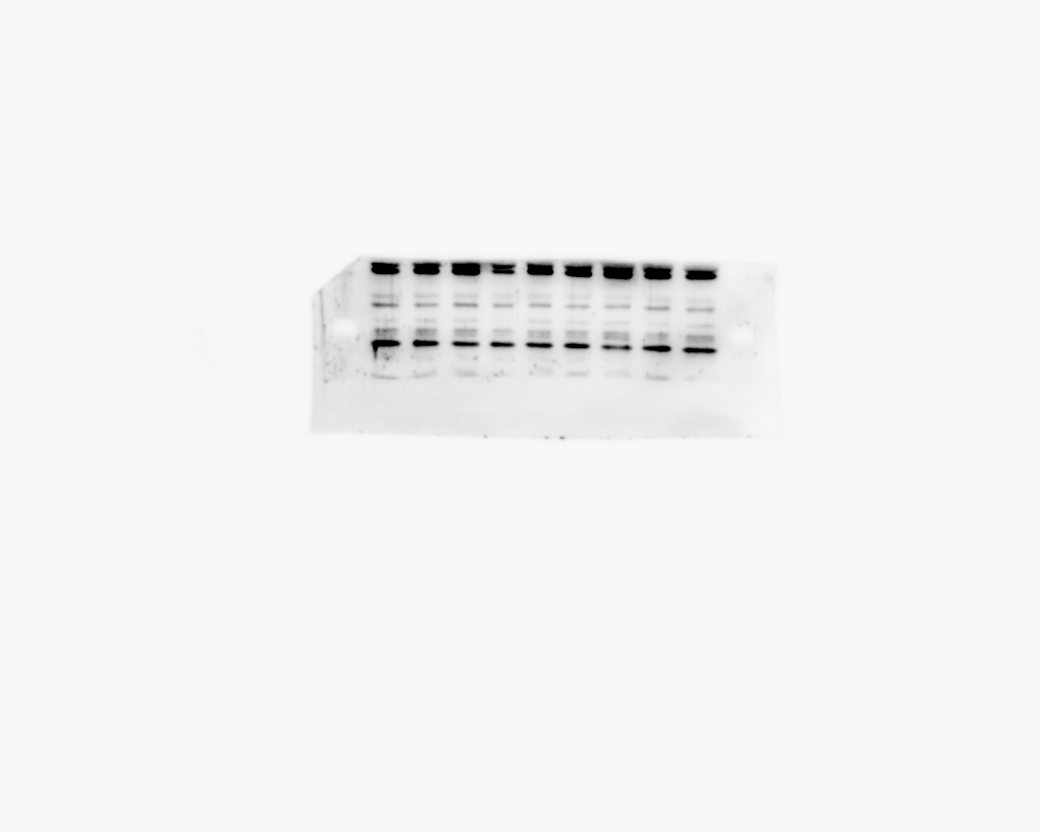


Figure 2C H3K27me3


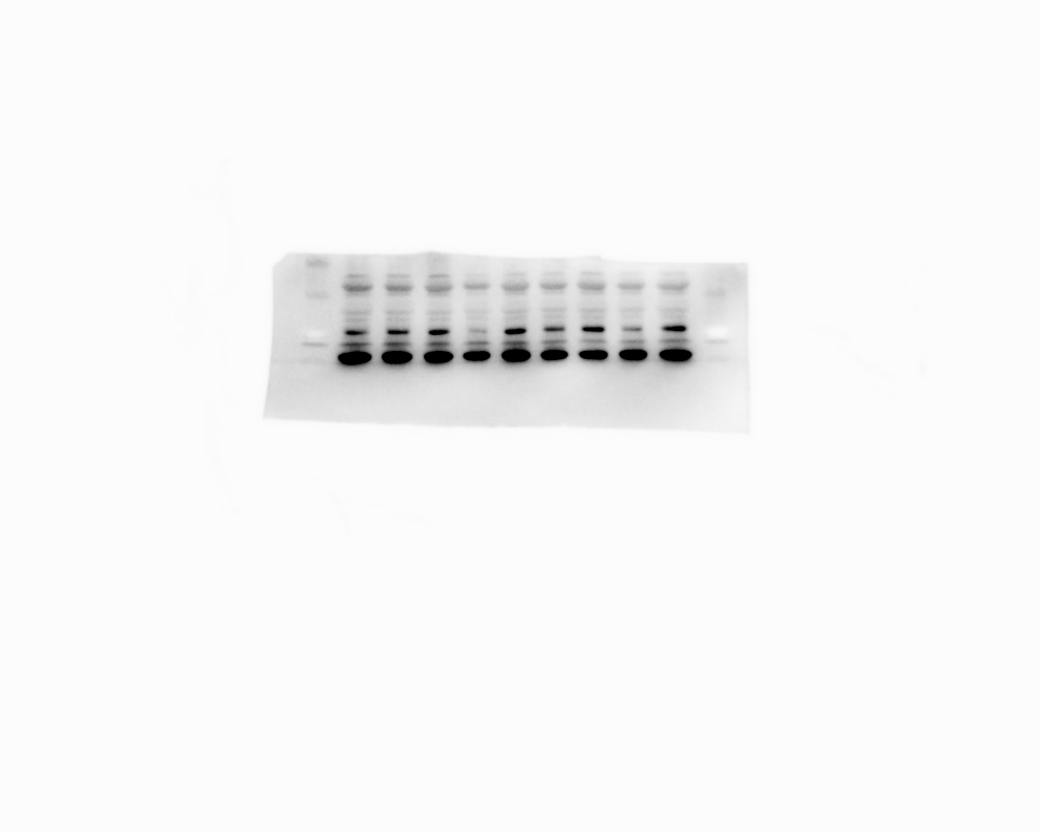


Figure 2C Total H3


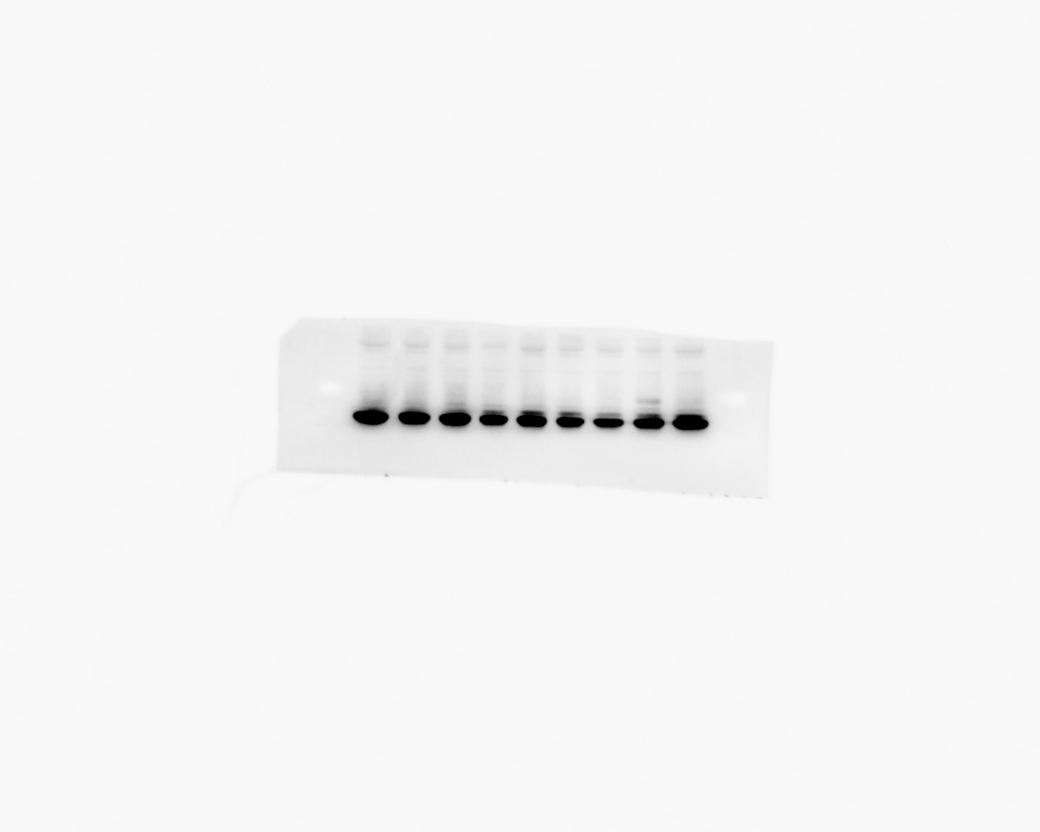

Supplement: Supplementary file 2 — Supplementary material 2. [file 42826_2025_254_MOESM2_ESM.docx]
