# Supplementary material for: Phosphorylation-mimicking histone H3.3 rescues exercise-induced gene responses in an epigenetic aging model of mouse skeletal muscle
Source: Lab Anim Res. 2025 Sep 24;41:25. doi: 10.1186/s42826-025-00254-6 (PMC12459025; doi:10.1186/s42826-025-00254-6)
Supplement: Supplementary file 3 — Supplementary material 3. [file 42826_2025_254_MOESM3_ESM.docx]

Matsumoto University Graduate School of Health Science

2095-1 Niimura, Matsumoto City, Nagano 390-1295, Japan

**Phone: +81-263-48-7372, FAX: +81-263-48-7290**

**e-mail: kawano@t.matsu.ac.jp**

July 30, 2025

Dear Editor,

Regarding our manuscript titled "Phosphorylation-mimicking histone H3.3 rescues exercise-induced gene responses in an epigenetic aging model of mouse skeletal muscle" (LARJ-D-25-00084R3), we are re-submitting the same set of files as in our previous submission. According to the previous decision letter, our manuscript was accepted pending proofreading, and we were informed that the proofreading staff would contact us regarding the necessary corrections. However, we have not received any communication from the editorial office or the proofreading staff. To address this, we contacted the editorial office twice during this period, but unfortunately, we have not received any further information. As today marks the deadline for the revision submission, we are re-submitting the previous version of our manuscript to fulfill the requirement. We would greatly appreciate it if you could kindly let us know what specific revisions are needed in the final version of the manuscript.

Thank you very much for your attention and kind support.

Sincerely,

Fumi Kawano, Ph.D.
